# Transthyretin Maintains Muscle Homeostasis through the Novel Shuttle Pathway of Thyroid Hormones during Myoblast Differentiation

**DOI:** 10.3390/cells8121565

**Published:** 2019-12-04

**Authors:** Eun Ju Lee, Sibhghatulla Shaikh, Dukhwan Choi, Khurshid Ahmad, Mohammad Hassan Baig, Jeong Ho Lim, Yong-Ho Lee, Sang Joon Park, Yong-Woon Kim, So-Young Park, Inho Choi

**Affiliations:** 1 Department of Medical Biotechnology, Yeungnam University, Gyeongsan 38541, Korea; gorapadoc0315@hanmail.net (E.J.L.); sibhghat.88@gmail.com (S.S.); apdltkd@naver.com (D.C.); ahmadkhursheed2008@gmail.com (K.A.); mohdhassanbaig@gmail.com (M.H.B.); lim2249@kitech.re.kr (J.H.L.); 2 Department of Biomedical Science, Daegu Catholic University, Gyeongsan 38430, Korea; ylee325@cu.ac.kr; 3 College of Veterinary Medicine, Kyungpook National University, Daegu 41566, Korea; psj26@knu.ac.kr; 4 Department of Physiology, College of Medicine, Yeungnam University, Daegu 42415, Korea; ywkim@yumail.ac.kr (Y.-W.K.); sypark@med.yu.ac.kr (S.-Y.P.)

**Keywords:** muscle satellite cell, transthyretin, thyroid hormone, myogenesis, exosomes, skeletal muscle

## Abstract

Skeletal muscle, the largest part of the total body mass, influences energy and protein metabolism as well as maintaining homeostasis. Herein, we demonstrate that during murine muscle satellite cell and myoblast differentiation, transthyretin (TTR) can exocytose via exosomes and enter cells as TTR- thyroxine (T_4_) complex, which consecutively induces the intracellular triiodothyronine (T_3_) level, followed by T_3_ secretion out of the cell through the exosomes. The decrease in T_3_ with the TTR level in 26-week-old mouse muscle, compared to that in 16-week-old muscle, suggests an association of TTR with old muscle. Subsequent studies, including microarray analysis, demonstrated that T_3_-regulated genes, such as FNDC5 (Fibronectin type III domain containing 5, irisin) and RXRγ (Retinoid X receptor gamma), are influenced by TTR knockdown, implying that thyroid hormones and TTR coordinate with each other with respect to muscle growth and development. These results suggest that, in addition to utilizing T_4_, skeletal muscle also distributes generated T_3_ to other tissues and has a vital role in sensing the intracellular T_4_ level. Furthermore, the results of TTR function with T_4_ in differentiation will be highly useful in the strategic development of novel therapeutics related to muscle homeostasis and regeneration.

## 1. Introduction

Skeletal muscle is comprised of multinucleated myofibers and has excellent regeneration capability, which deteriorates progressively with age, restraining the voluntary functions of daily life. The regenerative capacity is mostly facilitated by muscle satellite or stem cells (MSCs) that reside between the basal lamina and sarcolemma, a distinct ‘niche’ in the muscle fibers [1,2]. MSCs vigorously regulate myofiber growth, and MSC progression is typically regulated by the expression of myogenic transcription factors (Pax3, Pax7, myoblast determination protein; MYOD, and myogenin; MYOG) [3]. After injury, quiescent Pax7^+^ MSCs are triggered to undergo sequential activation, proliferation and differentiation involving MYOD, Myf5 and MYOG to generate multinucleated myotubes [4]. MSC differentiation is indispensable in the regeneration of skeletal muscle and is typically regulated by multiple signaling pathways and by the interaction of several extracellular matrix components with MSCs. Fibromodulin was reported to have a robust role in muscle regeneration by enhancing the recruitment of MSCs to injury sites [5].

Thyroid hormones (THs, thyroxin; T_4_ and triiodothyronine; T_3_) have vital roles in the development of various tissues, as well as in postnatal life, by modulating gene expressions [6,7]. THs regulate the expression of various proteins crucial for muscle development and contractility [8,9,10]. Indeed, the foremost targets of THs are muscles, as they regulate the expression of several genes at the transcriptional level [11,12]. The effects of TH signaling in the development and function of skeletal muscle are the result of a remarkably complex mechanism [11]. Generally, to retain homeostasis, regeneration capability, and development, binding of T_3_ to thyroid hormone receptors (TR) is essential [13]. TRs are encoded by two genes (THRA and THRB), and alternate splicing of each gene produces TRα1, TRβ1, and TRβ2 receptor subtypes. TRα is the predominant subtype in cardiac and skeletal muscle [14]. TRα has a key role in regulation of heart rate and basal metabolism [15]. Transcription of MYOD is directly regulated by T_3_ [16]. Therefore, TH signaling can control several events during myogenesis via direct and/or indirect regulation of myogenic gene expression.

Retinoids (synthetic vitamin A derivatives) can influence development and metabolism through nuclear hormone receptors (retinoic acid receptor and retinoid X receptor, RXR). RXR forms heterodimers with retinoic acid, TH, and vitamin D receptors, enhancing transcriptional function on their respective response elements [17]. Three different RXR isoforms (RXRα, β and γ) have been characterized. RXRγ is the dominant isoform in adult heart and skeletal muscle [18].

Exosomes are small (40–100 nm) membrane vesicles of endocytic origin that are released from most cell types into the extracellular environment [19]. Exosomes were first defined in 1983, and interest in these vesicles increased markedly after finding that they contain mRNA and microRNA [20]. Exosomes have been shown to facilitate cellular communication by transporting proteins, cytokines, and nucleic acids and to sustain the normal physiological function of cells [21].

Transthyretin (TTR) is a 55-kDa homotetrameric transporter protein for T_4_ and retinol-binding protein in the blood [22,23]. The liver is the main contributing organ for TTR synthesis in plasma. TTR null (TTR^−/−^) mice exhibit a delayed suckling-to-weaning transition, delayed growth, reduced muscle mass, and stunted longitudinal bone growth [24]. Among the transporters existing in blood, thyroxine-binding globulin (TBG) has the highest affinity for T_4_ and T_3_ (1.0 × 10^10^ and 4.6 × 10^8^ M^−1^, respectively), followed by TTR (7.0 × 10^7^ and 1.4 × 10^7^ M^−1^) and albumin (7.0 × 10^5^ and 1.0 × 10^5^ M^−1^) [25]. The binding efficacy of TH distributor proteins determines the transportation times for distribution of THs to tissues, thus, TTR (with transitional affinity), more than TBG, is responsible for instant delivery of THs to tissues [6,25].

Though it is known that human placenta, trophoblasts, JEG-3 and HepG2 cells secrete and internalize TTR [26,27,28], its cellular uptake in skeletal muscle has not been fully described. We have demonstrated that TTR initiates myoblast differentiation by inducing the expression of myogenic genes involved in the early phase of myogenesis and the associated calcium channels [6], and we have elucidated its functional role in maintaining the cellular T_4_ level. Furthermore, we reported that TTR enhances recruitment of MSCs to the site of injury, thereby regulating muscle regeneration [29]. However, the detailed mechanism of TTR with T_4_ in MSCs differentiation into muscle cells is unclear. In the current work, we have confirmed TTR secretion and internalization in myoblast cell. We found that TTR uptake and internalization by myoblast cells is increased by T_4_. By using microarray analysis and other studies, we have elucidated that TTR and TH coordinate with each other to modulate gene expression in muscle growth, development, and homeostasis.

## 2. Materials and Methods

### 2.1. Animal Experiments

C57BL/6 male mice were obtained from Daehan Biolink (Dae-Jeon, South Korea) and housed at four per cage in a temperature controlled room under a 12 h light/12 h dark cycle. In the period mice (six weeks) were fed a normal diet containing 4.0% (*w*/*w*) total fat (Rodent NIH-31 Open Formula Auto; Zeigler Bros., Inc., Gardners, PA, USA). Gastrocnemius muscle tissues were collected after 10 or 20 weeks. After collection, muscle tissues were fixed and stored at −80 °C until required for RNA and protein extraction or fixed overnight at 4 °C for paraffin-embedded tissue blocks to be used in immunohistochemistry. All experimental were done by following the guidelines issued by the Institutional Animal Care and Use Committees of the Catholic University of Daegu (IACUC-2017-051).

### 2.2. C2C12 Cell Culture

C2C12 cells (murine myoblast, Korean Cell Line Bank, Seoul, Korea) were cultured in DMEM (HyClone Laboratories, Logan, UT, USA) supplemented with 10% FBS (fetal bovine serum, HyClone Laboratories) and 1% P/S (penicillin/streptomycin, Thermo Fisher Scientific, Waltham, MA, USA) in a humidified 5% CO_2_ incubator at 37 °C. For differentiation, cells were cultured for two or three days in DMEM + 2% FBS + 1% P/S (serum (+) differentiation media) or DMEM + 1% P/S (serum (−) differentiation media). T_4_ (50 ng/mL, Sigma Aldrich, St. Louis, MO, USA), I-850 (5 ug/mL, Sigma Aldrich), TTR (0.1 ug/mL, Sigma Aldrich) or bovine serum albumin (BSA,1 mg/mL, Sigma Aldrich) was added to the indicated differentiation medium after two or three days.

### 2.3. Mouse MSCs Culture

Gastrocnemius and cranial thigh muscles were collected from C57BL male mice (six weeks) and minced, digested with 1% pronase (Roche, Mannheim, Germany) for 1 h at 37 °C, and then centrifuged at 1000× *g* for 3 min followed by passage of the digested tissue phase through a 100 mm syringe filter (Millipore, Darmstadt, Germany). After centrifugation of the filtrate at 1000× *g* for 5 min, the pellets were suspended in DMEM + 20% FBS + 1% P/S + 5 ng/mL FGF2 (fibroblast growth factor 2, Miltenyi Biotec GmbH, Auburn, CA, USA), seeded on collagen-coated plates (Corning, Brooklyn, NY, USA), and incubated in a humidified 5% CO_2_ atmosphere at 37 °C. The medium was changed every day. For induction of MSC differentiation into muscle cells, media were switched to DMEM + 2% FBS + 1% P/S or DMEM + 1% P/S followed by incubation for two days. MSC purity was confirmed with Pax7 protein expression (Santa Cruz Biotechnology, Paso Robles, CA, USA) using immunocytochemistry.

### 2.4. MTT Assay

C2C12 cells were cultured with DMEM + 10% FBS + 1% P/S for two days for analysis of cell viability. The cells were washed with DMEM and then incubated with 0.5 mg/mL MTT reagent (Sigma Aldrich) for 1 h. After dissolving the formazan crystals with DMSO (Sigma Aldrich), absorbance was measured at 540 nm (Tecan Group Ltd., Männedorf, Switzerland).

### 2.5. Immunoneutralization

TTR protein neutralization was carried out with TTR-specific antibodies (5 µg/mL, Santa Cruz Biotechnology) for two or three days in DMEM + 2% FBS + 1% P/S or DMEM + 1% P/S differentiation media.

### 2.6. Exosomes Isolation

Cells were cultured with DMEM + 1% P/S differentiation media. The cells were incubated for two or three days and the media were then collected, centrifuged at 2000× *g* for 30 min, and the upper phase collected for exosomes isolation. Using a total exosomes isolation reagent (Thermo Fisher Scientific, MA, USA), the exosomes from the upper phase were isolated according to the manufacturer’s protocol. In brief, the media were incubated with the total exosomes isolation reagent at 4 °C overnight and centrifuged at 10,000× *g* for 60 min. After discarding the supernatant, the pellet was dried at room temperature and suspended in PBS.

Mouse plasma (4 mL) was filtered with a 0.8 um syringe filter (Sartorius, Goettingen, Germany), and the exosomes were then isolated according to the manufacturer’s protocol (exoEasy Maxi Kit, Qiagen, Germantown, MD, USA).

### 2.7. T_4_ and T_3_ Concentration Measurement

An ELISA kit (DRG International, Marburg, Germany) was used to measure the concentration of T_4_ or T_3_ hormones. In brief, cell lysates or cultured media with T_4_ or T_3_ enzyme conjugate reagent were homogenized and added to specific antibody-coated microtiter plates and then incubated for 60 min at room temperature. After discarding the mixtures, the unbound materials were removed by washing the plates. Substrate solution was added followed by incubation for 20 min. Stop solution was then applied to terminate the reaction. Color intensities were then measured at 450 nm by using a spectrophotometer (Tecan Group Ltd., Switzerland).

### 2.8. Gene Knockdown

When C2C12 cells confluency reached 30%, 1 ng TTR, TR-α, RXRγ, or fibronectin type III domain containing 5 (FNDC5) shRNA vector (Santa Cruz Biotechnology) and scrambled vector (empty vector as negative control, Santa Cruz Biotechnology) were transfected using plasmid transfection reagent and transfection medium according to the manufacturer’s protocol (Santa Cruz Biotechnology). After three days, transfected cells were selected with puromycin (2 ug/mL, shRNA or scrambled vector is a puromycin selection vector, Santa Cruz Biotechnology). Selected cells were grown to 70% confluence before switching to differentiation media. Knockdown efficiencies were determined by analyzing the expressions of control (scrambled vector transfected cell) and knockdown cells. Supplementary Table S1 shows the sequences of the shRNA constructs.

### 2.9. RNA Isolation, cDNA Synthesis and RealTime RT-PCR

Trizol reagent (Thermo Fisher Scientific) was used following the manufacturer’s instructions to extract total RNA from cells. Two micrograms of RNA in 20 µL of reaction mixture was employed for the synthesis of 1st strand cDNA with random hexamer and reverse transcriptase at 25 °C for 10 min, 37 °C for 120 min, and 85 °C for 5 min. The cDNA product (2 µL) and gene-specific primers (10 pmole, 2 µL) were used for analysis of real-time RT-PCR (40 cycles), which was performed using a 7500 real-time PCR system with power SYBR Green PCR Master Mix (Thermo Fisher Scientific) as the fluorescence source. Glyceraldehyde 3-phosphate dehydrogenase (GAPDH) was used as the reference gene. Primer information is presented in Supplementary Table S2.

### 2.10. RT-PCR

Exosomes RNA was synthesized into cDNA and 2 µL cDNA and gene-specific primers (10 pmole, 2 µL) were used for PCR, which was performed using a 2720 Thermal Cycler PCR machine with PCR Master mix (Genetbio, Daejeon, Korea). The PCR conditions were as follow; denaturation 95 °C for 30 s, annealing at 59 °C for 30 s, extension at 72 °C, post-extension 72 °C for 5 min followed by holding (40 cycles). The PCR product was examined by performing electrophoresis on agarose gel.

### 2.11. Protein Isolation from Culture Media

The cells were cultured with DMEM + 1% P/S differentiation media for two days, centrifuged at 5000× *g* for 5 min, and the supernatant was then incubated with 1.3% potassium acetate (Sigma Aldrich) for 1 h at 4 °C. The mixture was centrifuged at 1500× *g* for 10 min, the supernatant was discarded, and the pellet washed with 100% acetone (Merck, Darmstadt, Germany). The isolated proteins were then dried, and Western blot analysis was performed by adding buffer with protease inhibitor cocktail (Thermo Fisher Scientific).

### 2.12. Western Blot

After washing the cells with PBS, they were lysed with RIPA buffer supplemented with protease inhibitor cocktail (Thermo Fisher Scientific). The Bradford assay was used to estimate the total protein concentration. Proteins (60 µg) were electrophoresed in 10% or 12% SDS-polyacrylamide gel and then transferred to PVDF membrane (EMS–Millipore, Billerica, MA, USA). The blots were then blocked with 3% skim milk or BSA in Tris-buffered saline (TBS)-Tween 20 for 1 h, incubated overnight with protein-specific primary antibodies [TTR (1:400), MYOD (1:500), MYOG (1:500), D2 (iodothyronine deiodinase type 2; 1:500), RXRγ (1:500) (Santa Cruz Biotechnology) or β-actin (1:2000) antibody (Santa Cruz Biotechnology), TR-α (1:500, Thermo Fisher Scientific), MYL2 (myosin light chain 2, 1:1000, Abcam, Cambridge, MA, USA) or FNDC5 (1:500, Bioss Antibodies, Woburn, MA, USA)] in 1% skim milk or BSA in TBS at 4 °C. The blots were then washed and incubated with horse radish peroxidase (HRP)-conjugated secondary antibody (Santa Cruz Biotechnology) for 1 h at room temperature and then developed with Super Signal West Pico Chemiluminescent Substrate (Thermo Fisher Scientific). Supplementary Table S3 shows the molecular weight of protein.

### 2.13. Fusion Index

After washing with PBS, cells were fixed with methanol and then stained with 0.04% Giemsa G250 (Sigma Aldrich). Images were taken randomly of three different sections per dish. The number of nuclei in myotubes and the total number of nuclei in the cells were counted in each field. Fusion indices were calculated by expressing the number of nuclei in the myotubes as percentages of the total numbers of nuclei.

### 2.14. TTR Protein Labeling with Fluorescence

TTR protein and BSA were labeled with the Alexa Fluor 594 protein labeling kit (Thermo Fisher Scientific) following the manufacturer’s instructions. Briefly, 100 µL TTR proteins and BSA (0.1 µg/µL) were incubated with 4.7 µL Alexa Fluor 594 succinimidyl ester (12.2 nmole/µL) for 15 min at room temperature, and the conjugated reaction mixture was then purified with resin gel-spin filter. Labeled TTR proteins (0.2 µg) and BSA were added to the cells and detected by fluorescence microscope (Nikon, Tokyo, Japan).

### 2.15. TTR Overexpression Vector 

The region corresponding to the TTR gene open reading frame (ORF) was PCR amplified with TTR ORF primer (Forward: 5′-ATGGCTTCCCTTCGACTCTTCC-3′, Reverse: 5′-GATTCTGGGGGTTGCTGACGA-3′) and ligated into the pcDNA 3.1/CT-GFP-TOPO vector (Invitrogen, Waltham, MA, USA). The ligated sequence was confirmed by sequencing analysis. The construct (2.5 µg) was transfected using 10 µL lipofectamine (1 mg/mL) and Opti MEM medium (Invitrogen) into C2C12 cells following the manufacturer’s directions and positive cells were selected using G418 antibiotics (2 µg/mL, AppliChem GmbH, Darmstadt, Germany). 

### 2.16. Immunocytochemistry

The cells were fixed with 4% formaldehyde (Sigma Aldrich) and permeabilized with 0.2% Triton X 100 (Sigma Aldrich). After blocking with 1% normal goat serum (SeraCare Life Sciences, Milford, MA, USA) for 30 min in a humid environment, cells were incubated with primary antibodies [TTR (1:50), MYOD (1:50), MYOG (1:50), MYL2 (1:50), D2 (1:50), RXRγ (1:50), TRα (1:50), or FNDC5 (1:50)] at 4 °C in a humid environment overnight. Secondary antibody (1: 100; Alexa Fluor 594 goat anti-rabbit or anti-mouse; Thermo Fisher Scientific) was applied for 1 h at room temperature. DAPI was used to stain the cells (Sigma-Aldrich) and imaged using a fluorescence microscope equipped with a digital camera (Nikon, Tokyo, Japan).

### 2.17. Immunohistochemistry

The sections of paraffin-embedded muscle tissue were deparaffinized and hydrated with xylene (Junsei, Tokyo, Japan) and ethanol (Merck), respectively, and endogenous peroxidase activity was quenched in 0.3% H_2_O_2_/methanol. The sections were then either stained with hematoxylin and eosin (Thermo Fisher Scientific) for morphological observation or blocked with 1% normal goat serum (SeraCare Life Sciences), incubated with primary antibodies [TTR (1:50), D2 (1:50), or FNDC5 (1:50)] overnight at 4 °C, and then incubated with horse radish peroxidase–conjugated secondary antibody (1:100). Positive signals were visualized by adding horse radish peroxidase-conjugated streptavidin (Vector, CA, USA). Nuclei of stained sections were stained with hematoxylin and then dehydrated, mounted, and observed by a light microscope (Leica, Wetzlar, Germany).

### 2.18. Microarray Analysis

Microarray analysis was conducted with the Agilent Technologies mouse GE4X 44K (V2) chip to determine the differentially expressed genes in wild-type (TTR_wt_) and TTR_kd_ (TTR knockdown) cells as described previously [30]. Briefly, TTR_wt_ and TTR_kd_ cells were grown in serum (+) differentiation media for two days and RNAs were extracted, synthesized into cDNA with fluorescence using a Low RNA Input Linear Amplification kit (Agilent Technologies, CA, USA) according to the manufacturer’s instructions. A total of three hybridizations were performed, and the statistical relevance of gene expression differences was confirmed by SAM (Standard University, Palo Alto, CA, USA). The significance cut-off was a median false discovery rate ≤5% for the SAM analysis.

### 2.19. DAVID Analysis

DAVID was performed as described previously [5]. In brief, enriched biological themes in up- and down-regulated gene lists (*p* ≤ 0.05 and 2 fold≤) were categorized by employing the Gene Ontology (GO) terms of cellular component, molecular function, and biological process in DAVID.

### 2.20. Statistical Analysis

Mean values of normalized expressions were evaluated by Tukey’s Studentized range test to categorize expressional differences of genes, considering *p* ≤ 0.05 statistically significant. The real-time RT-PCR data was normalized using glyceraldehyde 3-phosphate dehydrogenase (GAPDH) as the internal standard and was analyzed by one-way ANOVA using PROC GLM in SAS 9.0 (SAS Institute, Cary, NC, USA).

## 3. Results

### 3.1. TTR Secretion During Myoblast Differentiation

**Figure 1:**
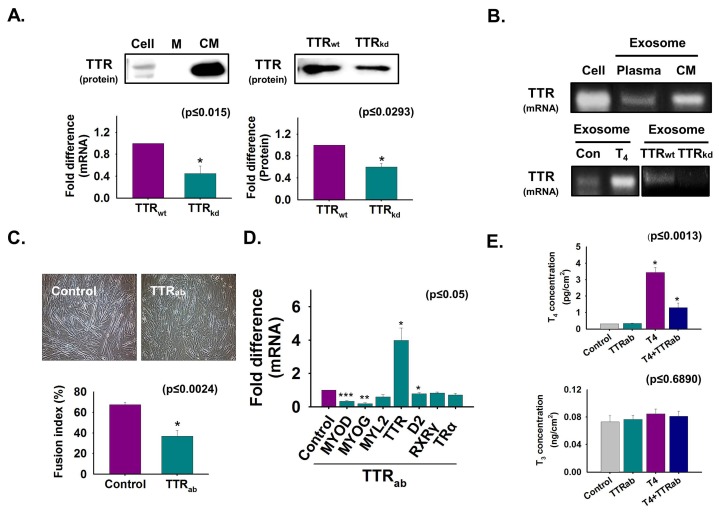
The role of secreted TTR from cells during myogenic differentiation. Normal and TTR knockdown cells were cultured with serum-free media for two days (**A**,**B**). (**A**) Proteins were isolated from cells, DMEM (control) and cultured media (CM). TTR protein level was analyzed by Western blot. TTR mRNA level in cells by real-time RT-PCR, and protein level in cell culture media of TTR_wt_ and TTR_kd_ by Western blot. Band intensity was measured by using ImageJ. (**B**) TTR mRNA levels in normal cell, exosomes isolated from mouse plasma, media of cultured C2C12 cells (CM) with or without T_4_ treatment, and TTR_wt_ and TTR_kd_ by RT-PCR. Cells were cultured in 2% FBS or serum-free media supplemented with TTR antibody for two (**C**) or three days (**D**,**E**) for immunoneutralization. (**C**) Myotube formation and fusion index was observed by Giemsa staining. (**D**) Gene expression was observed by real-time RT-PCR. (**E**) T_4_ and T_3_ concentration in cells was observed by ELISA. TTR_wt_ indicates cells transfected with scrambled vector. Means ± SD (*n* = 3). * *p* ≤ 0.05, ** *p* ≤ 0.001, *** *p* ≤ 0.0001.

To investigate TTR secretion from cells during C2C12 myoblast differentiation, normal and TTR knockdown cells were cultured in serum-free media for two days, after which the isolated protein level from cultured media was analyzed by Western blotting. The appearance of more TTR protein in cultured media compared to that in cell lysate indicates that TTR is secreted during myoblast differentiation (Figure 1A). Furthermore, TTR mRNA and protein were decreased in TTR_kd_ cells and cultured media, respectively, compared to those in TTR_wt_ cells (Figure 1A). Next, the TTR mRNA level was analyzed in normal cells and exosomes from mouse plasma and media of cultured cells (CM): T_4_-treated cells, TTR_wt_, and TTR_kd_. TTR mRNA was evident in exosomes isolated from culture media and mouse plasma and was increased by T_4_ treatment but decreased by TTR_kd_ (Figure 1B). TTR immunoneutralization using TTR antibody was performed during differentiation. Myotube formation and the expression of the myogenic genes were decreased by TTR neutralization. However, TTR expression was significantly enhanced in neutralized cells (Figure 1C,D). Interestingly, when the T_4_ concentration was measured in cells, it was higher in non-neutralized cells than in neutralized cells supplemented with T_4_ (Figure 1E). Taken together, these results show that TTR secreted from cells transported T_4_ into the cells during myoblast differentiation.

### 3.2. Enhancement of Myoblast Viability and Differentiation by TTR with T_4_

**Figure 2:**
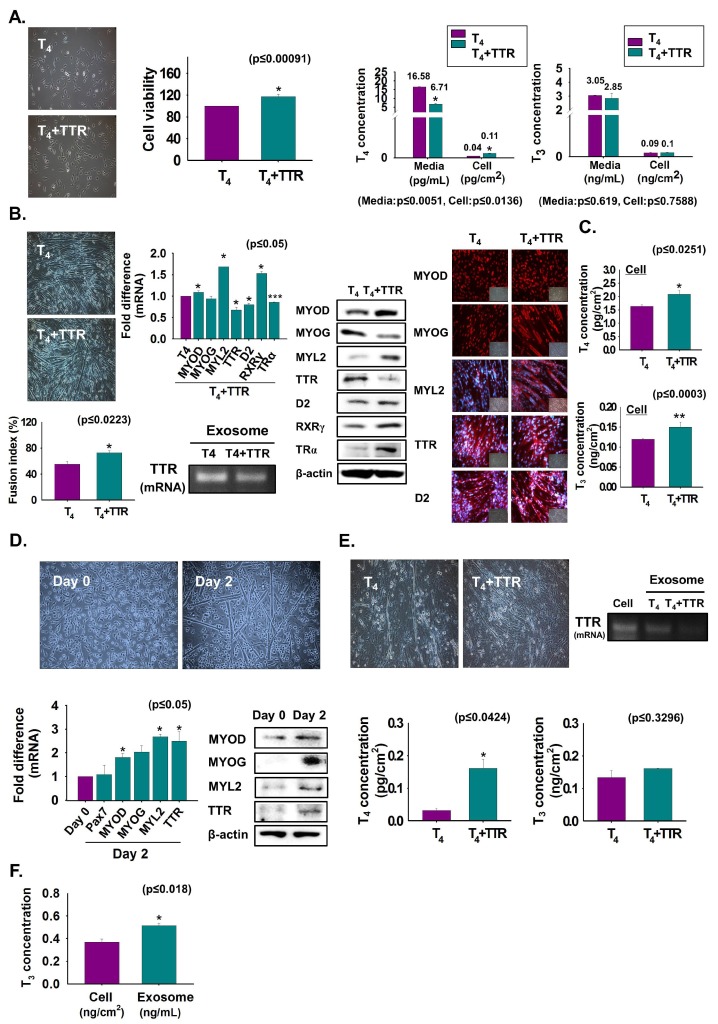
Myoblast viability and differentiation by treatment with TTR proteins. (**A**) C2C12 cells were cultured in 10% FBS media supplemented with T_4_ or T_4_ + TTR protein for two days. Cell viability was observed by MTT assay. T_4_ or T_3_ concentration in cultured media and cells were observed by ELISA. Cells were cultured in serum-free media supplemented with T_4_ or T_4_ + TTR protein for three days (**B**,**C**). (**B**) Myotube formation and fusion index by Giemsa staining, mRNA level in cells by real-time RT-PCR, exosomes by RT-PCR, protein expression by Western blot and immunocytochemistry. (**C**) T_4_ or T_3_ concentration in cells was observed by ELISA. (**D**) When mouse MSCs reached 100% confluency, media were switched to 2% FBS and cultured for zero and two days. MSC differentiation, TTR mRNA level by real-time RT-PCR and protein expression by Western blot. (**E**) MSCs were cultured in serum-free media supplemented with T_4_ or T_4_ + TTR protein for two days. T_4_ or T_3_ concentration in cells was observed by ELISA. (**F**) MSCs were cultured with serum-free media supplemented with T_4_ for two days and exosomes were isolated from cultured media. T_3_ concentration in cell and exosomes was observed by ELISA. Means ± SD (*n* = 3). * *p* ≤ 0.05, ** *p* ≤ 0.001, *** *p* ≤ 0.0001.

To assess the role of TTR and T_4_ on myoblast viability and differentiation, C2C12 cells were grown with T_4_ or T_4_ + TTR protein for two or three days. Cell viability was increased in T_4_ + TTR protein treated cells compared to that in only T_4_ treated cells (Figure 2A). The T_4_ and T_3_ concentrations were measured in CM and cells. A lower T_4_ concentration in media with a consequent higher concentration in cells was observed with T_4_ + TTR treatment than in those with only T_4_ treatment. The results indicate that TTR outside the cell enhances the transport of T_4_ to the cell interior in myoblast viability (Figure 2A). Further, cells were cultured in serum-free media with added T_4_ or T_4_ + TTR protein for three days to induce differentiation. The T_4_ + TTR treatment significantly induced myotube formation with elevated mRNA (MYL2) and protein expression of myogenic factors (MYOD and MYL2), RXRγ, and TRα. However, TTR mRNA and protein expression were decreased by TTR + T_4_ treatment and their expression in exosomes was also reduced from that of only T_4_-treated cells (Figure 2B). T_4_ and T_3_ concentrations were increased by TTR + T_4_ treatment (Figure 2C). TTR in mouse MSCs was assessed to determine its expression during differentiation. For this, MSCs were incubated with differentiation media for zero or two days. Expression of TTR and myogenic genes or proteins were increased on day 2 compared to that on day 0 (Figure 2D). Next, MSCs were cultured in serum-free conditions supplemented with T_4_ or T_4_ + TTR protein for two days to induce differentiation. Similar to the results with C2C12 cells, MSCs exhibited increased myotube formation with elevated thyroid hormone concentration under T_4_ + TTR treatment (Figure 2E). Interestingly, decreased TTR mRNA was observed in the exosomes following T_4_ + TTR treatment (Figure 2E). Furthermore, T_3_ was present in exosomes isolated from serum-free MSCs culture media supplemented with T_4_ (Figure 2F). These data showed that TTR protein with T_4_ not only enhanced myoblast proliferation and myogenic differentiation, but also increased MSC differentiation into muscle cells. 

### 3.3. Reduction of T_4_ Concentration Inside Cells and Myoblast Differentiation by Bovine Albumin Serum (BSA) Treatment

**Figure 3:**
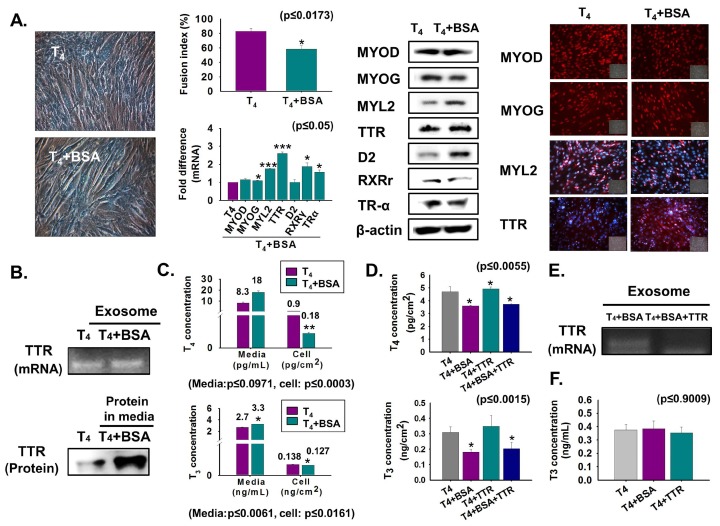
Myoblast differentiation following BSA treatment. Cells were cultured in serum-free media supplemented with T_4_ or T_4_ + BSA for two days (**A**–**E**). (**A**) Myotube formation and fusion index were observed by Giemsa staining. mRNA level was observed by real-time RT-PCR and protein expressions by Western blot and immunocytochemistry. (**B**) TTR mRNA in exosomes of cultured media using RT-PCR and protein level in cultured media by Western blot. (**C**) T_4_ or T_3_ concentration in cultured media or cells was observed by ELISA. (**D**,**E**) Cells were cultured with serum-free media supplemented with T_4_, T_4_ + BSA, T_4_ + TTR or T_4_ + BSA + TTR for two days. T_4_ or T_3_ concentration in T_4_ + BSA or T_4_ + BSA + TTR treated cells. TTR mRNA in exosomes of cultured media (in T_4_ + BSA or T_4_ + BSA + TTR treated cells) using RT-PCR. (**F**) Cells were cultured in serum-free media supplemented with T_4_ or T_4_ + BSA or T_4_ + TTR for two days and exosomes were isolated from each cultured medium. T_3_ concentration in exosomes. Means ± SD (*n* = 3). * *p* ≤ 0.05, ** *p* ≤ 0.001, *** *p* ≤ 0.0001.

For comparative assessment of T_4_ transport through TTR to the cell interior, C2C12 cells were cultured in serum-free media supplemented with T_4_ or T_4_ + BSA protein for two days. Myotube formation and MYOG expression were decreased in BSA-treated cells, while TTR and D2 expressions were increased at the translational level (Figure 3A). Interestingly, elevated TTR in both exosomes (mRNA) and CM (protein) was also observed in BSA-treated cells (Figure 3B). High T_4_ and T_3_ concentrations in T_4_ + BSA supplemented media with subsequent low levels in both hormone concentrations in the cell, under the same conditions, indicated that BSA reduced the transport of T_4_ to the cell interior (Figure 3C). Furthermore, elevated T_4_ concentration was observed in T_4_ + BSA + TTR supplemented cells relative to that in T_4_ + BSA treated cells (Figure 3D). Interestingly, decreased TTR mRNA was found in exosomes of T_4_ + BSA + TTR treated cells (Figure 3E). Additionally, T_3_ was present in exosomes, and there was no difference in T_3_ concentration in exosomes supplemented with T_4_, T_4_ + BSA, or T_4_ + TTR (Figure 3F). We observed that BSA reduces myotube formation by decreasing T_4_ transport. 

### 3.4. TTR Internalization Into Myoblast

**Figure 4:**
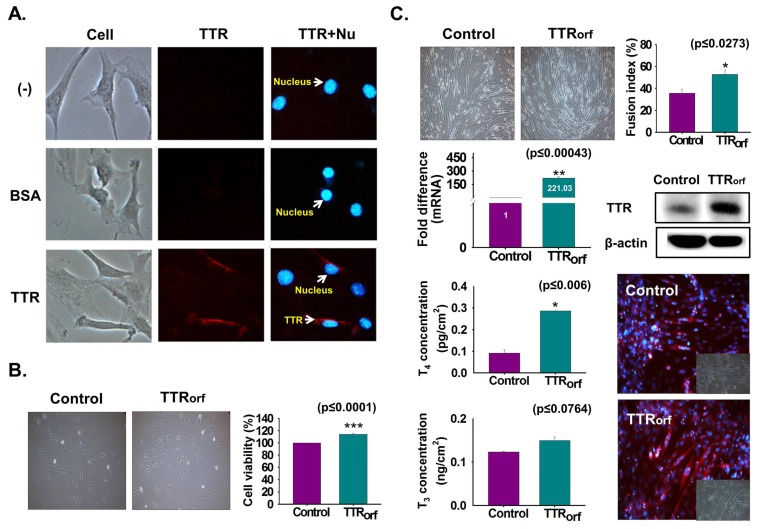
Endocytosis of TTR protein and TTR overexpression effects. (**A**) TTR protein or BSA were labeled with fluorescence and cells were cultured with serum-free media supplemented with labeled TTR protein or BSA for 1 day. Detection of labeled TTR protein and BSA in cells (Red: TTR, Blue: Nucleus). (**B**) TTR overexpression was performed by transfecting with TTR ORF plasmid followed by incubation with 10% FBS for two days. Cell viability was analyzed by MTT assay. (**C**) TTR overexpressing cells were incubated with serum-free media for two days. Myotube formation and fusion index were observed by Giemsa staining, TTR mRNA level by real-time RT-PCR, and protein expression by Western blot and immunocytochemistry. Control or TTR-overexpressing cells were incubated with serum-free media supplemented with T_4_ for two days. T_4_ or T_3_ concentration was measured by ELISA. Means ± SD (*n* = 3). * *p* ≤ 0.05, ** *p* ≤ 0.001, *** *p* ≤ 0.0001.

To elucidate TTR internalization to the cell interior, TTR protein or BSA was fluorescently labeled and C2C12 cells were cultured under serum-free conditions supplemented with labeled TTR protein or BSA for one day. Higher fluorescence of labeled TTR protein was evident in the cells treated with labeled TTR than with BSA or in non-treated cells (Figure 4A). TTR overexpression was achieved by transfection with the TTR ORF plasmid and cultured with 10% FBS for two days. Increased cell viability was observed in TTR-overexpressing cells (Figure 4B). Next, TTR-overexpressing cells were cultured with serum-free media for two days. Increased myotube formation with enhanced TTR mRNA/protein expression was observed in TTR-overexpressing cells (Figure 4C). Additionally, elevated concentrations of THs were observed in TTR-overexpressing cells supplemented with T_4_ (Figure 4C).

### 3.5. Regulation of RXRγ and TRα Expression by TTR During Myoblast Differentiation

**Figure 5:**
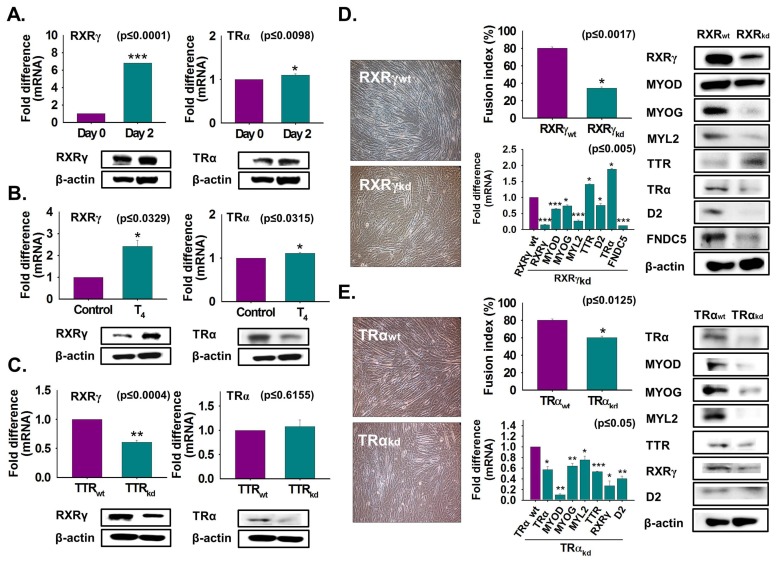
RXRγ and TRα expression during myoblast differentiation. (**A**) Cells were cultured with 2% FBS for two days. RXRγ and TRα expressions using real-time RT-PCR or Western blot. (**B**) Cells were cultured in serum-free media supplemented with T_4_ for two days. RXRγ and TRα expression by real-time RT-PCR or Western blot. (**C**) RXRγ and TRα expression in TTR_kd_ and TTR_wt_ cells using real-time RT-PCR or Western blot. (**D**) RXRγ knockdown was performed and followed by culture with 2% FBS for two days. Myotube formation and fusion index were observed by Giemsa staining, mRNA expression by real-time RT-PCR, and protein expression by Western blot in RXRγ_kd_ and RXRγ_wt_ cells. (**E**) TRα knockdown was performed and followed by culture with 2% FBS for two days. Myotube formation and fusion index were observed by Giemsa staining, mRNA expression by real-time RT-PCR, and protein expression by Western blot in TRα_kd_ and TRα_wt_ cells. TTR_wt_, RXRγ_wt_, or TRα_wt_ indicate cells transfected with the scrambled vector. Means ± SD (*n* = 3). * *p* ≤ 0.05, ** *p* ≤ 0.001, *** *p* ≤ 0.0001.

To determine the role of T_4_ or TTR on RXRγ and TRα expression, C2C12 cells were grown with or without serum in normal or TTR knockdown cells, and the effects were studied during myoblast differentiation. Increases in mRNA and protein expression of RXRγ and TRα were evident on day 2 compared to the levels on day 0 (Figure 5A). Next, T_4_ treatment under serum-free conditions stimulated RXRγ expression at both the transcriptional and translational level. However, TRα protein expression was decreased by T_4_ treatment (Figure 5B). Interestingly, TTR knockdown reduced expression of RXRγ and TRα (Figure 5C). Further, RXRγ and TRα knockdown were performed and followed by culturing with 2% FBS for two days. Myotube formation, myogenic genes and D2 expression were decreased by RXRγ or TRα knockdown, whereas TTR and TRα expressions were increased in RXRγ_kd_ cells. Most gene or protein expressions were decreased in TRα knockdown cells (Figure 5D,E). Overall, the above results indicate that expression of RXRγ is controlled by TTR via T_4_ transportation into the cell during myoblast differentiation.

### 3.6. Relationship between TTR and D2 According to Muscle Age

**Figure 6:**
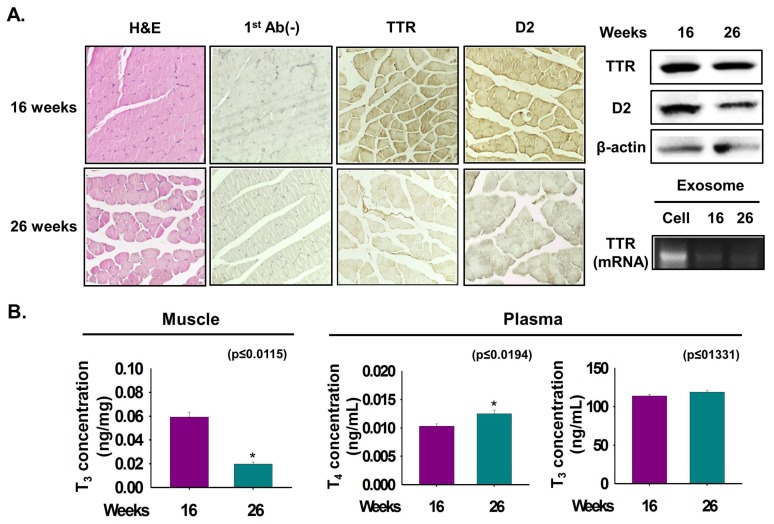
TTR expression and T_3_ concentration in age-dependent differences of muscle.. Expression of TTR and D2 proteins were analyzed in 16- and 26-week mouse muscles. (**A**) TTR and D2 proteins expression by Immunohistochemistry and Western blot. Exosomes were isolated from 16- and 26-week plasma. TTR mRNA level in cell and exosomes of 16- or 26-week plasma by RT-PCR. (**B**) T_3_ or T_4_ concentration in 16- or 26-week muscles or plasma was observed by ELISA. Means ± SD (*n* = 3). * *p* ≤ 0.05, ** *p* ≤ 0.001, *** *p* ≤ 0.0001.

To determine the effect of muscle age on TTR and D2 expression, mouse muscle at 16- and 26-weeks were collected. Myofiber size (width) and expression of TTR and D2 were decreased in 26-week muscle compared with 16-week muscle (Figure 6A). Interestingly, a decreased TTR level was observed in exosomes isolated from 26-week plasma (Figure 6A). The T_3_ concentration in 16-week muscle was higher than that in 26-week muscle (Figure 6B). Further, a significant increase in the T_4_ concentration in the plasma of 26-week mice the was observed, whereas there was no difference in the T_3_ concentration in the plasma of either age group (Figure 6B). The above findings suggest that expressions of TTR and D2 correlate with the age-dependent differences of muscle. 

### 3.7. Microarray Assessment of Gene Expression in TTR_kd_ Cells and Effect of T_4_ on Gene Expression

To explore TTR function in myoblast differentiation, TTR_kd_ and TTR_wt_ C2C12 cells were cultured with 2% FBS for two days. TTR/MYOG expression and myotube formation were decreased by TTR_kd_ (Supplementary Figure S1A,B). Microarray analysis was performed with TTR_wt_ and TTR_kd_ cells. After applying two-fold cut-offs for down- and up-regulated genes, analysis of the effects of knocking down TTR on myoblasts revealed that, among the genes involved in sarcomere formation, specific genes are actively up- or down-regulated, and some novel genes that are not involved in sarcomere formation functioned at the onset of myogenesis. Among the identified genes, 29 and 7 genes were down- or up-regulated, respectively, by greater than two-fold in TTR_kd_ cells (Table 1A,B; Supplementary Figure S1C,D). Many genes were previously reported to be involved in MSC maintenance (Heyl, Sox8), myogenesis (Fgf21, Ankrd2, Sox8, Asb2), proliferation (Ankrd2), myokine secretion (Fndc5), neuromuscular junction (Dok7), and Ca^2+^ release of sarcoplasmic reticulum (Asph). Even though some of these genes have identified roles in myogenesis, many novel genes were also affected by TTR_kd_ (R3hdml, Inpp4b, Igf2as, Btbd17, Sema6b and Ddc) (Table 1A; Supplementary Table S4A). However, the upregulated genes were mostly involved in the cell cycle, cell proliferation, and transcription regulation. Interestingly, there was little information indicating that those genes were related to muscle differentiation. Moreover, most of the up-regulated genes were novel genes, but their main functions have been studied in other tissues or organs (Table 1B; Supplementary Table S4B).

Down-regulated genes were analyzed at different myogenic times (0, 2, 4 and 6 days). Interestingly, most gene expressions were increased under myogenic conditions than that at the proliferating stage (Day 0). Similar to MYOG (the myogenic marker gene) the expression of 15 genes increased greatly during myogenic differentiation (Supplementary Figure S2). DAVID analysis was performed using the up- and down-regulated genes. More than half of the up-regulated genes were classified as transcription regulators (Table 1C), especially cell-cycle regulators. Although some of the down-regulated genes were identified as being involved in Ca^2+^-mediated signal transduction and were reported to regulate transcription, most down-regulated genes were classified as components or regulators of the sarcomere motor unit or the ATPase-related group, which are the main structural components of the sarcomere (Table 1D).

**Figure 7:**
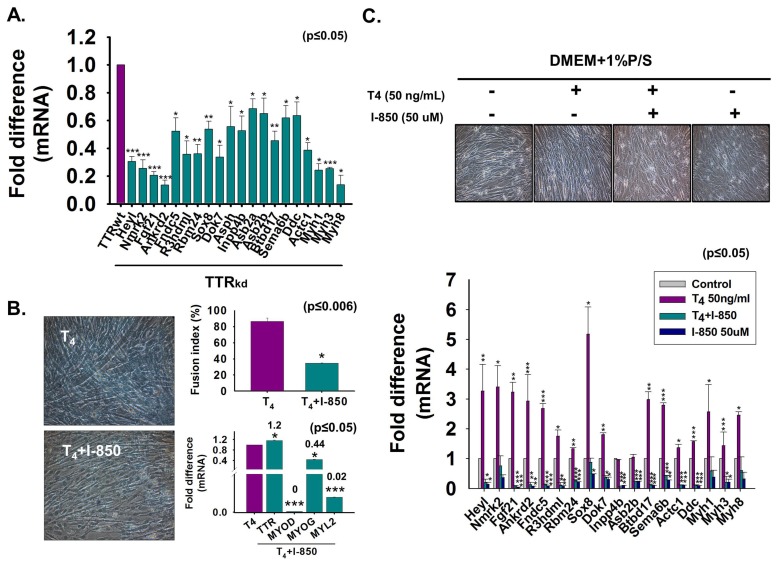
Expression of down-regulated genes in TTR knock-down cells and effect of T_4_ treatment on down-regulated genes. (**A**) TTR_wt_ or TTR_kd_ were cultured with 2% FBS for two days. Down-regulated gene expression was assessed by real-time RT-PCR in TTR_wt_ or TTR_kd_. (**B**) Cells were cultured with serum-free media supplemented with T_4_ or T_4_ + 1-850 and incubated for two days. Myotube formation and fusion index were observed by Giemsa staining and mRNA expression by real-time RT-PCR. (**C**) Cells were incubated without or with T_4_, T_4_ + 1-850 or 1-850 for two days. Expression of down-regulated genes without or with T_4_, T_4_ + 1-850 or 1-850 by real-time RT-PCR. Control indicates non-treated cells. Means ± SD (*n* = 3). * *p* ≤ 0.05, ** *p* ≤ 0.001, *** *p* ≤ 0.0001.

Even though genes were selected based on their high statistical significance among all differentially expressed genes, the genes were also cross-examined by performing real-time RT-PCR with TTR_kd_ and comparing the results to those of TTR_wt_ (Figure 7A). To investigate the effect of T_4_ on TR expression, cells were grown under serum-free conditions with added T_4_ and/or TR-specific antagonist 1-850 and examined both for morphological appearance and for changes in mRNA expression levels of certain genes. Myotube formation and mRNA levels of the myogenic marker genes (MYOD, MYOG and MYL2) were decreased by T_4_ + 1-850 treatment (Figure 7B). In contrast, T_4_ treatment increased myofibril diameter. The T_4_ treatment elevated most of the gene mRNA levels, whereas T_4_ + 1-850 treatment had opposite effects. However, T_4_ treatment reduced the suppressing effect of 1-850 on mRNA expression of Nmrk2 (40% rescue), Sox8 (40%), Myh1 (20%), and Myh8 (30%) (Figure 7C).

To determine whether the TTR_kd_ effects were produced by TH and its specific receptor, the TR binding site was scanned in genome portions containing the 5′ flanking region and the first intron of each gene. For precise analysis, two nuclear receptor scanning software programs, NHR-scan and NUBI-scan, were utilized. All binding site candidates were predicted by using the AGGTCA sequence arranged by the DR0, DR4, IR0, IR4, ER4, and ER6 patterns, as was used in the in silico thyroid hormone response elements (TRE) prediction models. Consequently, most of the genes contained more than one TRE at the 5′ flanking region. However, some genes such as Fgf21 did not have a suitable TRE. In addition, Myh1 did not possess a TRE upstream of the first exon (Supplementary Figures S3 and S4). Altogether, these results showed that T_4_ transported to the cell interior activated TR to induce gene expression and modulated novel and major transcription regulating genes that markedly increased during myogenic differentiation in a TH-dependent manner.

### 3.8. FNDC5 Expression During Myoblast Differentiation

**Figure 8:**
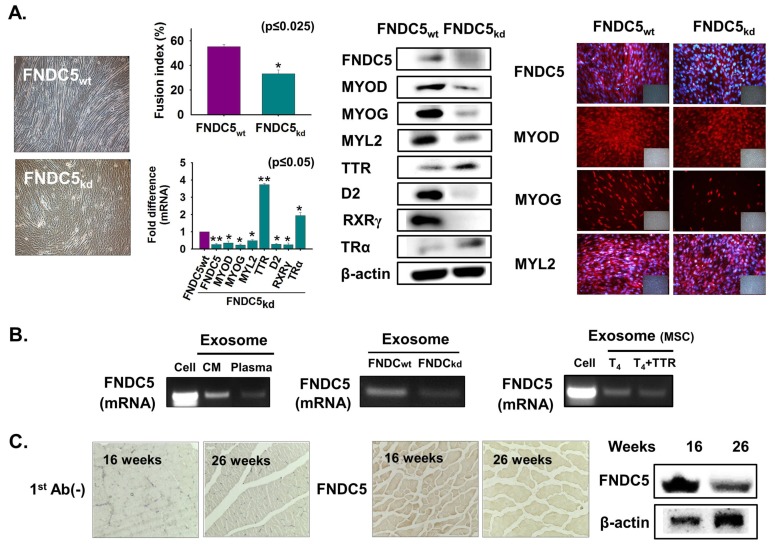
FNDC5 expression during myoblast differentiation. (**A**) FNDC5 knockdown was performed and cells were incubated with 2% FBS for two days. Myotube formation and fusion index were observed by Giemsa staining, mRNA expression using real-time RT-PCR and protein expression were observed by Western blot and immunocytochemistry. (**B**) Cells were cultured with only serum-free media for two days and exosomes were isolated from cultured media. FNDC5 mRNA level in normal cells, exosomes isolated from plasma, and media of cultured cells (FNDC5_wt_ and FNDC5_kd_). MSCs were cultured with only serum-free media or supplemented with T_4_ for two days. FNDC5 mRNA level in exosomes from cell, media of cultured cells with T_4_ or T_4_ + TTR. (**C**) FNDC5 protein expression in 16- or 26-week muscle by immunohistochemistry and Western blot. FNDC5_wt_ indicates cells transfected with scrambled vector. Means ± SD (*n* = 3). * *p* ≤ 0.05, ** *p* ≤ 0.001, *** *p* ≤ 0.0001.

To confirm the function of the genes that were down-regulated by TTR_kd_, myokine FNDC5 was selected. FNDC5 knockdown was performed followed by culture with 2% FBS for two days. Myotube formation and myogenic gene expression were decreased in FNDC5_kd_ cells, whereas TTR and TRα expressions were increased at both the transcriptional and translational levels (Figure 8A). Next, cells were grown in serum-free media or supplemented with T_4_ for two days, and the FNDC5 mRNA level was analyzed in normal cells and exosomes from plasma and media of cultured cells (FNDC5_kd_ and FNDC5_wt_). FNDC5 mRNA was evident in exosomes from culture media and plasma and decreased in FNDC5_kd_ cells (Figure 8B). Additionally, decreased FNDC5 mRNA was observed in T_4_ + TTR treatment in MSCs exosomes (Figure 8B). Expression of FNDC5 was decreased in 26-week muscle compared with that in 16-week muscle (Figure 8C). These results show that FNDC5 positively regulates myoblast differentiation. 

## 4. Discussion

Skeletal muscle accounts for nearly half of the body mass and represents the largest protein reservoir in the human body [31]. Although the importance of TH signaling in muscle physiology has been documented for several years, its precise mechanism in skeletal muscle during postnatal myogenesis remains unclear. Initially, we demonstrated the role of TTR in sustaining the cellular T_4_ level during myoblast proliferation and differentiation [6,29]. In this study, we give the first direct evidence of TTR secretion and uptake in C2C12 mouse myoblast cells. We also identify TTR mRNA in exosomes and its increased expression following T_4_ treatment, which may act as a mediator in this process. In addition, we studied the role of TTR in T_4_ transport into C2C12 cells and murine MSCs during the assessment of cell viability and differentiation. The appearance of TTR in cultured serum-free media from myoblasts strongly suggests that TTR synthesized by C2C12 cell is secreted. This suggestion was confirmed by TTR immunoneutralization using TTR antibody, which demonstrated a reduction in myotube formation and mRNA level of some myogenic marker genes (especially, MYOD and MYOG), and T_4_ uptake into cells, along with an increase in TTR retained in the cells. Further, it is important to emphasize the presence of T_3_ in exosomes, which indicates that T_3_ produced in cells is secreted out of the cell through exosomes. These results imply that muscle may not only utilize T_4_ but also act as a reservoir of T_3_ in order to distribute it to other tissues or to more distant sites. 

Cosmo et al. reported that TH uptake by skeletal muscle can occur independently of monocarboxylate transporter 8 (Mct8). However, they found enhanced TH action, T_3_ content, and glucose metabolism in Mct8 knockout mice [32]. We speculate that TTR might maintain the TH content in Mct8 knockout mice and, hence, normal muscle metabolism and development. The binding affinity of TTR for T_4_ is high, hence, it serves as a primary distributor protein in muscle. We showed that TTR with T_4_ treatment significantly increased cell viability and differentiation compared to that of only T_4_ treated cells. This was consistent with our previous finding that TTR expression increases myoblast differentiation by increasing T_4_ transport into the cell [29]. Similar to what we observed in the C2C12 cell line, the T_4_ + TTR protein treated mouse MSCs also showed increased myotube formation with elevated T_4_ concentration. Additionally, a progressive increase in TTR expression was observed during differentiation (day 2) in primary MSC cultures. These data confirm that TTR promotes myogenesis by enhancing the transport of T_4_.

Kassem et al. showed that the availability of TTR in cerebrospinal fluid (CSF) was associated with enhanced T_4_ uptake into the choroid plexus and brain and this uptake was increased in the presence of TTR [33]. Accordingly, in the present study, low T_4_ concentration in media with its consequent high concentration in cells supplemented with T_4_ + TTR indicated that TTR enhanced the transport of T_4_ to the cell interior during myoblast viability. The enhanced cell uptake may be a simple consequence of the increased T_4_ level in serum, providing a concentration gradient that promotes TTR secretion and subsequent cell uptake. Furthermore, increased uptake of TH in cells treated with both T_4_ and TTR probably involves a T_4_ complex with TTR, as well as passive diffusion of T_4_, allowing for greater cell uptake than can be accomplished by diffusion only, which is consistent with observations in human ependymoma cells [34]. Although TTR has been reported to be the main component in maintaining high TH levels in CSF and brain [33,35], in this study we observed that TTR also sustained the TH concentration in skeletal muscle and, hence, promoted myogenesis.

TTR is one of three proteins required for T_4_ transport: TBG is the major transporter and albumin has the lowest affinity, acting as the third T_4_ binding protein in human plasma [25,36]. Consistent with this theory, we found that BSA reduced T_4_ transport to the cells, which was also increased with TTR treatment as it has high efficiency for TH. Additionally, BSA treatment decreased myotube formation and myogenic protein expression, while TTR and D2 expressions were increased at the translational level, which might reflect the drop in the T_4_ level in the cells. TH regulates several genes that are responsible for muscle development and homeostasis. Among those genes, MYOD, MYOG and contractility-determining proteins are transcriptionally regulated by TH and are important for regeneration and myogenesis [37]. MYOD expression regulated by TH is involved in the fast muscle fiber phenotype, with transcriptional stimulation of the myosin-1, myosin-2 and myosin-4 isoforms [38]. TH metabolizing enzyme D2 can activate TH by outer-ring deiodination and can influence local tissue TH levels [39]. Collectively, our findings suggest that TTR acts to maintain the TH level in myoblast cells.

Evidence of high fluorescence-labeled TTR protein levels in cells reveals that TTR was internalized into the myoblast cells. This supports previous results showing endocytosis of fluorescence-labeled TTR in ependymoma cells [34]. In other reports, ^125^I-TTR and digoxigenin labeled TTR were internalized by an endocytic process in rat yolk sac and β-cells, respectively [40,41]. Furthermore, increased uptake of T_4_ in TTR-overexpressing cells supplemented with T_4_ implies that an even distribution of T_4_ within the cell is not only dependent on the free fraction of T_4_ in serum but also on the T_4_ bound to TTR. The presence of T_4_ or T_3_ significantly enhanced TTR internalization in JEG-3 cells, with TTR entering the cells as a TTR-T_4_ complex [27]. In addition, Divino and Schussler [42] reported increased TTR internalization in HepG2 cells with increasing amounts of T_4_ and suggested that a T_4_-stimulated conformational alteration in TTR somehow enhanced the uptake of TTR.

Reduced T_4_ serum concentrations have been reported in old rats [43,44], though their serum T_3_ level remains more controversial [43]. We show that TTR and D2 expressions with T_3_ concentration have a correlation with muscle age. The reduced D2 activity is suggestive of impaired T_4_ conversion in 26-week muscle. Silvestri et al. observed reduced D1 activity in 26-month-old rats relative to that in young (6- and 12-month-old male) rats [44]. Interestingly, decreased TTR expression in 26-week muscle was consistent with the decreased TH transporter Mct8 protein level in liver of 24-month-old rats [44]. Furthermore, higher plasma T_3_ or T_4_ concentration in 26-week muscle could be associated with the reduced free T_4_ concentration in the 16-week muscle, probably due to a higher TBG expression, as described elsewhere [45]. Additionally, decreased T_3_ concentration in the 26-week muscle at the cellular level was consistent with the findings of Silvestri et al. [44] in which decreased T_3_ concentration was observed in 24-month-old rats. Nevertheless, T_3_ generation has been observed in 11-month-old rats relative to that in seven-month-old rats [46], indicating that the mechanisms of T_3_ production from T_4_ in old muscle remain poorly understood.

TH is the main endocrine regulator that acts by binding to TRs and imposing a signature type of gene expression [11]. TH primarily functions either via nuclear receptor-mediated stimulation that is T_3_ dependent or by switching off the gene transcription machinery [13]. In muscle, this signaling pathway is regulated by the THRA1 isoform of TR [47]. The heterodimer complex formed by the TR with RXR- binds to a TRE, leading to activation or suppression of gene transcription [13]. Accordingly, we showed that T_4_ treatment induced RXRγ expression. However, myotube formation and myogenic factors were decreased in RXRγ and TRα knockdown cells. Interestingly, RXRγ knockout mice are unable to increase their mass in response to high-fat feeding, suggesting a specific effect of RXRγ in skeletal muscle [48]. In muscle, the proteins whose expression are transcriptionally controlled by T_3_ are SERCA1a [12], SERCA2a [49], uncoupling protein 3 (UCP3) [50], GLUT4 [51], cytosolic malic enzyme (ME1) [52], muscle glycerol-3-phosphate dehydrogenase (mGPDH) [53], and myosin-7 [54]. Furthermore, we found that TTR and D2 expression were decreased in TRα_kd_ cells, which explains the retarded transport of TH into the cell. The selective functions of TRs are controlled by local ligand availability [39,55] or by TH transport to the cell interior via Mct8 or other associated transporters [56]. The TH metabolizing enzymes D2 and D3, as well as transporters Mct8 and Mct10, are expressed in both rodent and human skeletal muscle [57,58].

The TTR-affected genes identified by TTR_kd_-based microarray analysis included important transcription factors or mediators that have the potential to control several other genes. For example, Rbm24 is reported to regulate MYOG expression [59] and mediate skeletal muscle-specific splicing events [60]. In contrast, Sox8, a negative regulator of myogenesis [61], has increased expression during myogenesis of C2C12 cells. In addition, Sox8 and Heyl genes are marker genes of MSCs [61,62]; however, the Heyl gene showed increased expression during myogenic differentiation. Interestingly, those opposing results were also observed for Nmrk2 [63]. Another research group reported that Ddc is not produced by myotubes [64], but in the present study, it was induced by suitable myogenic differentiation. Altogether, some genes that have been reported to be negatively correlated with myogenesis were markedly increased in expression during myogenic differentiation in this study.

Another interesting observation from the time-course expression study is that several novel genes that show increased expression during myogenesis responded to T_4_ as they did to TTR. However, Inpp4b and Asb2, genes that contain TREs in proximity to the transcription start site (TSS), did not show any change with T_4_ treatment. In the case of Inpp4b, TREs in the proximity of the promoter were only downstream of the TSS, and the first intron was approximately 130 kb. This characteristic indicates a rare aspect of the TTR_kd_-affected genes. Moreover, Fgf21 and Myh1, which do not seem to contain TREs, showed increased expression levels. The various TRE elements have only been predicted by a one-dimensional arrangement, moreover, a proper, precise, and complete nucleotide matrix for this one-dimensional arrangement is not present in public databases. Due to these limitations, many other researchers [65,66] have reported different nucleotide matrices for TREs and different reactivity of each.

Interaction and cooperation between TR and the mammalian insulator CCCTC-binding factor have already been reported [67,68]. An insulator can mediate multi-dimensional chromosomal changes [69]. In addition, based on the results of the TTR_kd_ microarray analysis, the T_4_ affected sarcomere genes Myh1, Myh3 and Myh8 may be suitable candidates for TR-insulator mediated transcriptional regulation. In the case of the Myh1 gene, no TREs were present in its promoter region.

In contrast, the FNDC5 gene, downregulated by TTR_kd_, also showed a high expression level during myogenic differentiation and after T_4_ treatment. The FNDC5 gene encodes the irisin protein, which is considered as a circulating myokine. The most remarkable feature of the FNDC5/irisin protein is that it generates brown fat from white fat [70,71]. Recently, it has been shown that irisin injection stimulated muscle hypertrophy and increased regeneration in injured skeletal muscle [72]. Additionally, enhanced irisin levels have been found during myogenic differentiation and the additional irisin enhances the expression of p-Erk, which has a vital role in the protein synthesis pathway [73]. Thus, knockdown of the FNDC5 gene was undertaken. We showed that interruption of the FNDC5 gene produced a low level of myotube formation. In humans, FNDC5 protein is cleaved to provide detectable irisin levels in circulation. Additionally, increased irisin concentrations occur in response to exercise in humans [74]. Therefore, based on the pro-myogenic role of FNDC5 in the present study, we suggest that FNDC5 may be a potential curative target for the intrusion of muscle dystrophy. Thus, we conclude that one control pathway within TTR myogenesis is mediated by the protein FNDC5.

## 5. Conclusions

**Figure 9:**
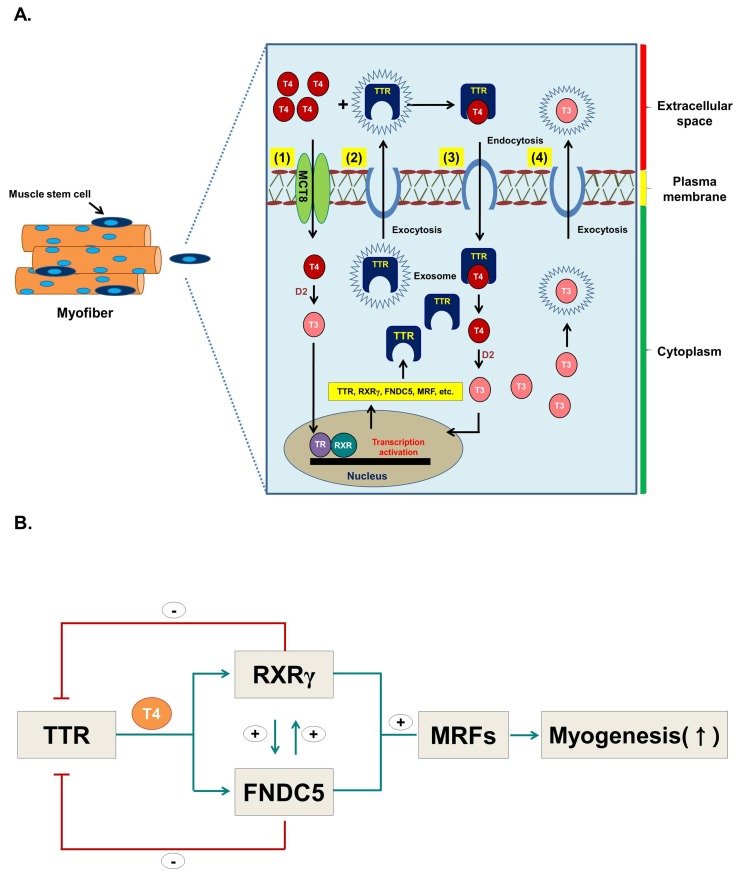
Hypothesis for the role of TTR with T_4_ during myoblast differentiation. (**A**) Hypothetical figure depicting role of TTR with T_4_ during myoblast differentiation. (1) T_4_ enters cells via Mct8 by passive diffusion and is converted to T_3_ by D2 enzyme, which in turn triggers the expression of several genes including TTR. (2) Synthesized TTR is exocytosed through exosomes, and (3) subsequently enters the cells as TTR-T_4_ complex via an endocytic mechanism. (4) T_3_ produced in the cells can exocytose via exosomes. (**B**) TTR positively regulates RXRγ and FNDC5 and triggers myogenic regulatory factors, hence promoting myogenesis. RXRγ and FNDC5 negatively regulate TTR while RXRγ and FNDC5 regulate each other.

In conclusion, these results suggest that: (1) a portion of the extracellular T_4_ enters myoblasts or myocytes via MCT via passive diffusion and is converted to T_3_ by the D2 enzyme which, in turn, induces the expression of several genes including TTR; (2) synthesized TTR exocytoses the cell through exosomes; (3) TTR brings T_4_ inside the cells as a TTR-T_4_ complex through an endocytic mechanism; (4) intracellularly synthesized T_3_ can exocytose via exosomes (Figure 9A); and (5) TTR, through the action of T_3_ converted from T_4_, regulates gene expression of TTR intermediates, such as RXRγ and FNDC5 (irisin), which ultimately induces myogenesis (Figure 9B). In this study, we have shown that muscle cells use a much more active mechanism than previously thought to bring T_4_ into cells. Moreover, intracellularly-generated T_3_, besides being used in the target muscle cells, also moves out of the cell and affects adjacent cells as well as probably other tissues. Herein, we propose a novel mechanism for the uptake and release of T_4_ and T_3_ in myoblasts and for TTR to act as a sensor for intracellular T_4_ during myogenesis. However, this study has presented a most rudimentary picture of T_4_ and T_3_ transport into and out of muscle cells, and further studies will undoubtedly reveal more detailed mechanisms.

## Figures and Tables

**Table 1:** Microarray analysis of TTR knockdown.

**A.**									
**Gene**	**Set1**	**Set2**	**Set3**	**Set4**	**Average**	***p* Value**	**Description**		
Myh1	0.16	0.31	0.13	0.06	0.16	0.0001	Mus musculus myosin, heavy polypeptide 1, skeletal muscle, adult (Myh1)		
Heyl	0.25	0.19	0.15	0.1	0.17	0.0001	Mus musculus hairy/enhancer-of-split related with YRPW motif-like (Heyl)		
Myo18b	0.17	0.4	0.13	0.05	0.19	0.0001	Mus musculus myosin XVIIIb (Myo18b)		
Myh8	0.2	0.39	0.12	0.08	0.2	0.0001	Mus musculus myosin, heavy polypeptide 8, skeletal muscle, perinatal (Myh8)		
Nmrk2	0.16	0.37	0.06	0.22	0.2	0.0001	Mus musculus nicotinamide riboside kinase 2 (Nmrk2)		
Fgf21	0.14	0.35	0.06	0.32	0.22	0.0001	Mus musculus fibroblast growth factor 21 (Fgf21)		
Ankrd2	0.17	0.36	0.06	0.28	0.22	0.0001	Mus musculusankyrin repeat domain 2 (stretch responsive muscle) (Ankrd2)		
Myom3	0.18	0.49	0.11	0.11	0.22	0.0001	Mus musculusmyomesin family, member 3 (Myom3)		
Myh3	0.18	0.39	0.14	0.21	0.23	0.0001	Mus musculus myosin, heavy polypeptide 3, skeletal muscle, embryonic (Myh3)		
Myh7	0.3	0.33	0.12	0.2	0.24	0.0001	Mus musculus myosin, heavy polypeptide 7, cardiac muscle, beta (Myh7)		
Myh3	0.21	0.4	0.15	0.21	0.24	0.0001	Mus musculus myosin, heavy polypeptide 3, skeletal muscle, embryonic (Myh3)		
Fndc5	0.44	0.26	0.18	0.1	0.25	0.0001	Mus musculus fibronectin type III domain containing 5 (Fndc5)		
R3hdml	0.35	0.4	0.09	0.15	0.25	0.0001	Mus musculus R3H domain containing-like (R3hdml)		
Myh7b	0.31	0.39	0.07	0.22	0.25	0.0001	Mus musculus myosin, heavy chain 7B, cardiac muscle, beta (Myh7b)		
Rbm24	0.2	0.41	0.15	0.23	0.25	0.0001	Mus musculus RNA binding motif protein 24 (Rbm24)		
Sox8	0.45	0.21	0.13	0.22	0.25	0.0001	Mus musculus SRY (sex determining region Y)-box 8 (Sox8)		
Dok7	0.21	0.5	0.08	0.29	0.27	0.0002	Mus musculus docking protein 7 (Dok7)		
Tnnt1	0.31	0.37	0.12	0.29	0.27	0.0001	Mus musculus troponin T1, skeletal, slow (Tnnt1), transcript variant 1		
Ttn	0.17	0.48	0.24	0.2	0.27	0.0001	Mus musculus titin (Ttn), transcript variant N2-B		
Asph	0.28	0.46	0.18	0.16	0.27	0.0001	Mus musculus aspartate-beta-hydroxylase (Asph), transcript variant 8		
Inpp4b	0.2	0.41	0.33	0.16	0.27	0.0001	Mus musculus inositol polyphosphate-4-phosphatase, type II (Inpp4b)		
Igf2os	0.37	0.45	0.15	0.16	0.28	0.0001	Mus musculus insulin-like growth factor 2, opposite strand (Igf2os), antisense RNA		
Myh6	0.42	0.23	0.18	0.32	0.28	0.0001	Mus musculus myosin, heavy polypeptide 6, cardiac muscle, alpha (Myh6)		
Asb2	0.48	0.39	0.23	0.17	0.32	0.0001	Mus musculusankyrin repeat and SOCS box-containing 2 (Asb2)		
Mybpc1	0.45	0.44	0.25	0.13	0.32	0.0001	Mus musculus myosin binding protein C, slow-type (Mybpc1)		
Btbd17	0.47	0.45	0.1	0.29	0.33	0.0002	Mus musculus BTB (POZ) domain containing 17 (Btbd17)		
Sema6b	0.46	0.39	0.09	0.38	0.33	0.0002	Mus musculussema domain, transmembrane domain (TM), and cytoplasmic domain		
Actc1	0.38	0.39	0.33	0.25	0.34	0.0001	Mus musculus actin, alpha, cardiac muscle 1 (Actc1)		
Ddc	0.48	0.48	0.22	0.39	0.39	0.0001	Mus musculusdopa decarboxylase (Ddc), transcript variant 1		
**B.**									
**Gene**	**Set1**	**Set2**	**Set3**	**Set4**	**Average**	***p*** **Value**	**Description**		
Gm10536	4.95	7.99	11.97	13.93	9.71	0.0049	Mus musculus predicted gene 10536 (Gm10536), long non-coding RNA		
Iws1	3.92	4.7	2.15	10.28	5.26	0.5130	Mus musculus IWS1 homolog (S. cerevisiae) (Iws1)		
Dkk2	4.02	3.54	4.95	2.98	3.87	0.0005	Mus musculusdickkopf homolog 2 (Xenopuslaevis) (Dkk2)		
Cdc45	3.44	2.22	4.92	3.16	3.43	0.0048	Mus musculus cell division cycle 45 (Cdc45), transcript variant 1		
Suv420h1	3.25	2.86	2.71	3.45	3.07	0.0001	Mus musculus suppressor of variegation 4–20 homolog 1 (Drosophila) (Suv420h1)		
Cdc42bpa	2.28	2.11	3.11	4.32	2.95	0.0082	Mus musculus CDC42 binding protein kinase alpha (Cdc42bpa)		
Zfp318	2.02	2.89	2.23	4.18	2.83	0.0094	Mus musculus zinc finger protein 318 (Zfp318), transcript variant 2		
**C.**									
**Term**							**Count**	**%**	***p* Value**
Transcription regulation							3	42.9	7.5 × 10^−2^
Nucleus							4	57.1	9.8 × 10^−2^
**D.**									
**Term**							**Count**	**%**	***p* Value**
Muscle protein							8	28.6	1.40 × 10^−13^
Thick filament							6	21.4	1.00 × 10^−12^
Myosin							7	25	1.40 × 10^−11^
Motor protein							7	25	5.90 × 10^−9^
Actin-binding							6	21.4	8.50 × 10^−6^
ATP-binding							10	35.7	1.20 × 10^−5^
Calmodulin-binding							5	17.9	1.80 × 10^−5^
Methylation							6	21.4	6.60 × 10^−5^
Nucleotide-binding							10	35.7	8.90 × 10^−5^
Coiled coil							11	39.3	1.40 × 10^−3^
Cytoplasm							10	35.7	4.80 × 10^−2^
Isopeptide bond							4	14.3	9.40 × 10^−2^

TTR_wt_ or TTR_kd_ were cultured with 2% FBS for two days and microarray analysis was performed on TTR_wt_ or TTR_kd_. (**A** and **B**) List of down- or up-regulated genes in TTR_kd_ (2-fold≤). (**C** and **D**) Functional analysis by DAVID (2-fold≤). TTR_wt_ indicates cells transfected with scrambled vector. Means ± SD (*n* = 3).

## Supplementary Materials

The following are available online at https://www.mdpi.com/2073-4409/8/12/1565/s1, Figure S1: Microarray analysis of TTR_kd_ cells, Figure S2: Time-course study of down-regulated genes during myoblast differentiation, Figure S3: Promoter of down-regulated genes was analyzed to predict TRE binding site, Figure S4: Promoter of down-regulated genes was analyzed to predict TRE binding site, Table S1: shRNA information, Table S2: Primer information, Table S3: Molecular weight of protein, Table S4: Functional analysis of up- or down-regulated genes affected by TTR_kd_.

## Abbreviations

TTR	Transthyretin
RXR	Retinoid X receptor
MSCs	Muscle satellite cells
MYOG	Myogenin
T_4_	Thyroxin
T_3_	Triiodothyronine
THs	Thyroid hormones
TR	Thyroid hormone receptors
MYOD	Myoblast determination protein
TBG	Thyroxine binding globulin
FBS	Fetal bovine serum
P/S	Penicillin/Streptomycin
D2	Iodothyronine deiodinase type 2
TRE	Thyroid hormone response elements
Mct8	Monocarboxylate transporter 8
TSS	Transcription start site
CSF	Cerebrospinal fluid
BSA	Bovine serum albumin
FNDC5	Fibronectin type III domain containing 5
CM	Culture media MYL2 (Myosin light chain 2)
