# Supplementary material for: Transthyretin Maintains Muscle Homeostasis through the Novel Shuttle Pathway of Thyroid Hormones during Myoblast Differentiation
Source: Cells. 2019 Dec 4;8(12):1565. doi: 10.3390/cells8121565 (PMC6952784; doi:10.3390/cells8121565)
Supplement: Supplementary file 1 [file cells-08-01565-s001.pdf]

Transthyretin Maintains Muscle Homeostasis through Novel Shuttle Pathway of Thyroid Hormone during Myoblast Differentiation

Eun Ju Lee[1#], Sibhghatulla Shaikh[1#], Dukhwan Choi[1], Khurshid Ahmad[1], Mohammad Hassan Baig[1], Jeong Ho Lim[1], Yong-Ho Lee[2], Sang Joon Park[3], Yong-Woon Kim[4], So-Young Park[4] & Inho Choi[1]*

[1]Department of Medical Biotechnology, Yeungnam University, Gyeongsan 38541, Korea

[2]Department of Biomedical Science, Daegu Catholic University, Gyeongsan, 38430, Korea

[3]College of Veterinary Medicine, Kyungpook National University, Daegu, 41566, Korea

[4]Department of Physiology, College of Medicine, Yeungnam University, Daegu 42415, Korea

[#]These two authors contributed equally to this work

***Corresponding Author:**

Prof. Inho Choi
Department of Medical Biotechnology
Yeungnam University
Gyeongsan, 38541, Republic of Korea
Fax: +82 53 810 4769
E-mail: inhochoi@ynu.ac.kr

Table S1. shRNA information

shRNA	Sequence		
TTR	TTR shRNA Plasmid (m) is a pool of 3 different shRNA plasmids		
	SC-39716-SHA	Hairpin sequence	GATCCCTGTAGACGTGGCTGTAAATTCAAGAGATTTACAGCCACGTCTACAGTTTTT
		Sense	CUGUAGACGUGGCUGUAAAtt
		Antisense	UUUACAGCCACGUCUACAGtt
	SC-39716-SHB	Hairpin sequence	GATCCGAAGATGCCGTGAAGCATTTTCAAGAGAAATGCTTCACGGCATCTTCTTTTT
		Sense	GAAGAUGCCGUGAAGCAUUtt
		Antisense	AAUGCUUCACGGCAUCUUCtt
	SC-39716-SHC	Hairpin sequence	GATCCCACCTGCTATTTCATTCAATTCAAGAGATTGAATGAAATAGCAGGTGTTTTT
		Sense	CACCUGCUAUUUCAUUCAAtt
		Antisense	UUGAAUGAAAUAGCAGGUGtt
RXRγ	RXRγ shRNA Plasmid (m) is a pool of 3 different shRNA plasmids		
	sc-60868-SHA	Hairpin sequence	GATCCCCATCAGGAAAGATCTCATTTCAAGAGAATGAGATCTTTCCTGATGGTTTTT
		Sense	CCAUCAGGAAAGAUCUCAUtt
		Antisense	AUGAGAUCUUUCCUGAUGGtt
	sc-60868-SHB	Hairpin sequence	GATCCGAACGTGGAGAACTCAACATTCAAGAGATGTTGAGTTCTCCACGTTCTTTTT
		Sense	GAACGUGGAGAACUCAACAtt
		Antisense	UGUUGAGUUCUCCACGUUCtt
	sc-60868-SHC	Hairpin sequence	GATCCCTTCGACAGAGTCCTTACATTCAAGAGATGTAAGGACTCTGTCGAAGTTTTT
		Sense	CUUCGACAGAGUCCUUACAtt
		Antisense	UGUAAGGACUCUGUCGAAGtt
TRα	TRα shRNA Plasmid (m) is a pool of 3 different shRNA plasmids		
	sc-43689-SHA	Hairpin sequence	GATCCCTACCGCTGTATCACTTGTTTCAAGAGAACAAGTGATACAGCGGTAGTTTTT
		Sense	CUACCGCUGUAUCACUUGUtt
		Antisense	ACAAGUGAUACAGCGGUAGtt
	sc-43689-SHB	Hairpin sequence	GATCCCCTGATACATGTTGCTACATTCAAGAGATGTAGCAACATGTATCAGGTTTTT
		Sense	CCUGAUACAUGUUGCUACAtt
		Antisense	UGUAGCAACAUGUAUCAGGtt
	sc-43689-SHC	Hairpin sequence	GATCCGCAAGGGGAGAATATAGATTTCAAGAGAATCTATATTCTCCCCTTGCTTTTT
		Sense	GCAAGGGGAGAAUAUAGAUtt
		Antisense	AUCUAUAUUCUCCCCUUGCtt
FNDC5	FNDC5 shRNA Plasmid (m) is a pool of 3 different shRNA plasmids		
	sc-143012-SHA	Hairpin sequence	GATCCCCTATCATCGGGCTTATTATTCAAGAGATAATAAGCCCGATGATAGGTTTTT
		Sense	CCUAUCAUCGGGCUUAUUAtt
		Antisense	UAAUAAGCCCGAUGAUAGGtt

	sc-143012-SHB	Hairpin sequence	GATCCGTCAGCTTGGATAACTGAATTCAAGAGATTCAGTTATCCAAGCTGACTTTTT
		Sense	GUCAGCUUGGAUAACUGAAtt
		Antisense	UUCAGUUAUCCAAGCUGACtt
	sc-143012-SHC	Hairpin sequence	GATCCCCTTCATTCGGGTCTCTAATTCAAGAGATTAGAGACCCGAATGAAGGTTTTT
		Sense	CCUUCAUUCGGGUCUCUAAtt
		Antisense	UUAGAGACCCGAAUGAAGGtt

Table S2. Primer information

Species	Gene	Product size (bp)	Tm (°C)	Sequence (F)	Sequence (R)
Mouse	GAPDH	155	59	5'-tgctggtgctgagtatgtcg-3'	5'-caagcagttggtggtacagg-3'
Mouse	TTR	165	59	5'-tggacaccaaatcgtactgg-3'	5'-aattctgggggttgctgac-3'
Mouse	MYOG	185	59	5'-tccagtacattgagcgccta-3'	5'-caaatgatctcctgggttgg-3'
Mouse	MYOD	213	59	5'-aggagcacgcacacttctct-3'	5'-tctcgaaggcctcattcact-3'
Mouse	MYL2	177	59	5'-aaagaggctccaggtccaat-3'	5'-cctctctgcttgtgtggtca-3'
Mouse	RXRγ	190	59	5'-cctacggtgacatgaacgtg-3'	5'-gagaaggaggcaatgagcag-3'
Mouse	TRα	259	59	5'-tgacattggccagtcaccta-3'	5'-ctcagggtcagggtgtcact-3'
Mouse	D2	152	59	5'-gatgctcccaattccagtgt-3'	5'-caggtggctgaaccaaagtt-3'
Mouse	Heyl	164	56	5′-agaccgcatcaacagtagcc-3′	5′-ctcgggcatcaaagaacc-3′
Mouse	Nmrk2	146	57	5′-acagccaacgctacttcctg-3′	5′-ctgctccatctcccgtctat-3′
Mouse	Fgf21	165	55	5′-agatcagggaggatggaaca-3′	5′-caaagtgaggcgatccatag-3′
Mouse	Ankrd2	143	58	5′-accaacgtgagagacaagctac-3′	5′- gcatcatgtagggcactgtc-3′
Mouse	Fndc5	175	59	5′-agtgagcctgtgctcttcaa-3′	5′- ggcggcagaagagagctataa-3′
Mouse	R3hdml	142	57	5′-ggtctgcaactacgccatt-3′	5′- gagcctgttggatttgagc-3′
Mouse	Rbm24	153	57	5′-ctttggcgttcaacagcttc-3′	5′- tcaatgtacggtgtggtgga-3′
Mouse	Sox8	168	57	5′-atgtcacacgtggaggattc-3′	5′-ggcaccagactccagtcata-3′
Mouse	Dok7	132	57	5′-caatctgtggcggactcaag-3′	5′-catacgtgggcatctcactg-3′
Mouse	Asph	183	58	5′-tggtgggttagccaagagga-3′	5′-gatgctttctgggatgtgtct-3′
Mouse	Inpp4b	175	56	5′-gatgtcggtgacactggaac-3′	5′-gtgtcgagaagttggcttgt-3′
Mouse	Asb2a	170	56	5′-ggaggccaagaatgtctacag-3′	5′-ctaccacgtcttcatgctca-3′
Mouse	Asb2b	167	56	5′-ccatcggacatgaggaataca-3′	5′-ggtccatgggtggaaatgt-3′
Mouse	Btbd17	153	57	5′-gcaaccagagtgaggttatgc-3′	5′-aggatgctactcggtacttgg-3′
Mouse	Sema6b	163	56	5′-cacagtaaagcacgactccaa-3′	5′-cacgtcattcttgcacacac-3′
Mouse	Actc1	163	56	5′-tctggcgatggtgtaactca-3′	5′-caatttcacgttcagcagtgg-3′
Mouse	Ddc	110	56	5′-tgcaggcttacatccgaaag-3′	5′-gcagaccaacccaagaatga-3′
Mouse	Myh1	122	53	5′-ctctctggaacaagaaaagaaaatc-3′	5′-caagctgctgtttatcgttttctac-3′
Mouse	Myh3	130	57	5′-gttaagccagaggacgtgtatg-3′	5′-tcatccaggacgtgtatcgg-3′
Mouse	Myh8	219	56	5′-agtaatgcagaggccatcg-3′	5′-cttgctccttgacaactgagag-3′

Table S3. Molecular weight of protein

Protein	Molecular weight (kDa)
β-actin	43-44
TTR	17
MYOD	45
MYOG	34
MYL2	19
D2	31
RXRγ	50-54
TRα	48
FNDC5	22

Table S4.

Supplementary Table 1 Functional analysis of up- or down-regulated genes affected by TTR_{kd}. Function or role in myogenesis of each **(A)** downregulated and **(B)** upregulated genes. Listed functions were derived from literature review, gene database searches, etc. Rate indicates the fold change average of four independent sample sets of microarray analysis of TTR_{wt} or TTR_{kd}.

A. TTR_{kd} microarray down-regulated gene information related to muscle

Gene	Full name	Rate	Function & Highlight	Etc.
Heyl (Hesr3)	Hairy/enhancer-of-split related with YRPW motif-like	0.17	• Satellite cell fate decision<br>• Downstream target : Nrf2	• Size : 35kDa<br>• bHLH Transcription factor (binds to E box : CACGTG)
Nmrk2 (MIBP)	Nicotinamide riboside kinase 2	0.2	• Reduces laminin matrix deposition and cell adhesion<br>• Interaction : Integrin α7β1 (skeletal muscle specific integrin)	• Size : 22kDa<br>• Kinase of nicotinamide riboside

Fgf21	Fibroblast growth factor 21	0.22	• Promotes myoblast differentiation<br>• Switch anaerobic myofibers to aerobic myofibers<br>• Controls glucose uptake<br>• Upstream positive controller : MYOD, thyroid hormone, insulin-Akt signaling	• Size : 22kDa<br>• Endocrine FGF (FGF 15/19, FGF21, FGF 23)
Ankrd2 (AARP)	Ankyrin repeat domain 2	0.22	• Promotes myoblast differentiation<br>• Controls apoptosis and proliferation<br>• Upstream positive controller : MYOD, Nkx2.5, p53<br>• Interaction : Titin (I-band)	• Size : 40kDa / 36kDa<br>• Muscle Ankyrin repeat<br>• Protein (Ankrd1, Ankrd2, Ankrd23)
Fndc5 (PeP)	Fibronectin type III domain-containing protein 5	0.25	• Myokine (Irisin)<br>• Controls mitochondria function<br>• Upstream positive controller : Smad3<br>• Downstream target : IGF-1, MSTN, PPARγ	• Post translation modification :<br>• 212 aa → 112 aa<br>• Circulating hormone
R3hdml	R3H Domain Containing Like	0.25	• Muscle regeneration	• Size : 28kDa<br>• Putative Sserine protease inhibitor
Rbm24	RNA Binding Motif Protein 24	0.25	• Controls muscle specific splicing events<br>• Critical controller of sarcomerogenesis<br>• Upstream positive controller : MYOD<br>• Downstream target : MYOG, MYOD	• Size : 25kDa<br>• Nuclear protein
Sox8	SRY (sex determining region Y)-box 8	0.25	• Satellite cell marker<br>• Negative regulator of skeletal muscle differentiation<br>• Downstream target : basic helix-loop-helix transcription factors	• Size : 47kDa<br>• Transcription factor (binds on [AT] CAA [AT])
Dok7	Docking protein 7	0.27	• Neuromuscular junction formation.<br>• Acetylcholine receptor (AchR) accumulation	• Size : 53kDa / 73kDa / 28kDa<br>• Non-catalytic cytoplasmic adaptor
Asph	Aspartate-beta-hydroxylase	0.27	• Ca2+ storage and release of sarcoplasmic reticulum<br>• Skeletal muscle specific : Junctin (Variant 8)	• Size: 34kDa (of 86kDa)<br>• ER membrane Ca^{2+} sensor
Inpp4b	Inositol polyphosphate 4-phosphatase type II	0.27	• PIP signaling pathway<br>• Highly expressed isoform at skeletal muscle	• Size : 105kDa / 15kDa<br>• PIP signaling phosphatase
Igf2os (Igf2as)	Insulin-like growth factor 2, opposite strand	0.28	• Highly expressed at myoblast<br>• May encode protein sequence	• Long non-coding RNA<br>• Overlaps partially with IGF2 gene
Asb2	Ankyrin repeat and SOCS box-containing 2	0.32	• Skeletal muscle specific : Asb2b<br>• Controls myogenic differentiation<br>• Upstream negative controller : TGF-β	• Size : 70kDa<br>• Ankyrin repeat sequenceand SOCS box domain
Btbd17	BTB (POZ) domain containing 17	0.33	• No data	• Size : 52kDa<br>• Secretion protein

Sema6b	Sema domain, transmembrane domain, and cytoplasmic domain, 6B	0.33	• Upstream negative controller : PPARα	• Size : 95kDa / 74kDa<br>• Transmembrane protein
Actc1	Actin, alpha, cardiac muscle 1	0.34	• Cardiogenesis marker<br>• 5% of total skeletal muscle actin<br>• Predominant at fetal skeletal muscle	• Size : 42kDa<br>• Cytoskeletal protein
Ddc	Dopa decarboxylase; Nmrk2, nicotinamide riboside kinase 2	0.39	• Converts levodopa to dopamine<br>• Not presented in myotube	• Size : 54kDa / 37kDa / 45kDa<br>• Dopamine convertase

B. TTR_{kd} microarray up-regulated gene information related to muscle

Gene	Full name	Rate	Function & Highlight	Etc.
Gm10536	Predicted gene 10536 (Gm10536), long non-coding RNA	9.71	• Long non-coding RNA (Predicted gene)<br>• No data	
Iws1	IWS1 homolog (S. cerevisiae)	5.26	• Cell survival and proliferation<br>• Interaction : RNA polymerase II	• Size : 92kDa / 56kDa / 69kDa<br>• RNA Pol II elongation factor
Dkk2	Dickkopf homolog 2	3.87	• High expression level at quiescent satellite cell<br>• Upstream positive controller : MSTN<br>• Upstream negative controller : APC<br>• Interaction : Wnt signaling	• Size : 28kDa<br>• Secretion protein<br>• Agonist & Antagonist :<br>• Wnt/β-catenin signaling
Cdc45	Cell division cycle 45 (Cdc45), transcript variant 1	3.43	• Upstream negative controller : MYOG	• Size : 66kDa / 60kDa / 69kDa<br>• Initiator of DNA replication
Suv420h1 (=KMT5B)	Suppressor of variegation 4-20 homolog 1	3.07	• Support to maintain quiescence satellite cell property<br>• Heterochromatin formation	• Size : 99kDa / 45kDa / 32kDa<br>• Histone H4K20me3 transferase
Cdc42bpa	CDC42 binding protein kinase alpha	2.95	• No data	
Zfp318	Zinc finger protein 318	2.83	• Androgen receptor modulation	

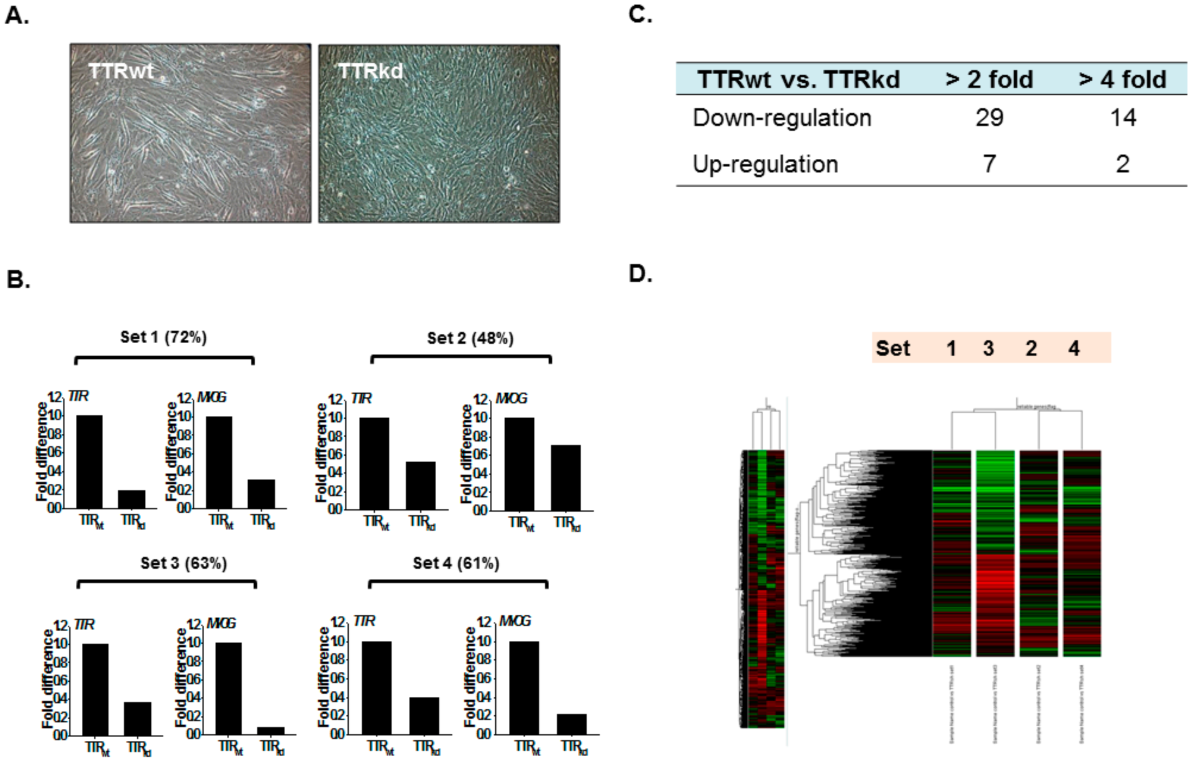

Supplementary Fig. 1. Microarray analysis of TTRkd cells. TTRwt or TTRkd were cultured with 2% FBS for 2 days. **A)** Myotube formation in TTRwt and TTRkd. **B)** TTR and MYOG level by real-time RT-PCR in four sets of TTRwt and TTRkd cells. **C)** The number of down- and up regulated genes (p≤0.05, 2- or 4-fold ≤) **D)** Hierarchical cluster

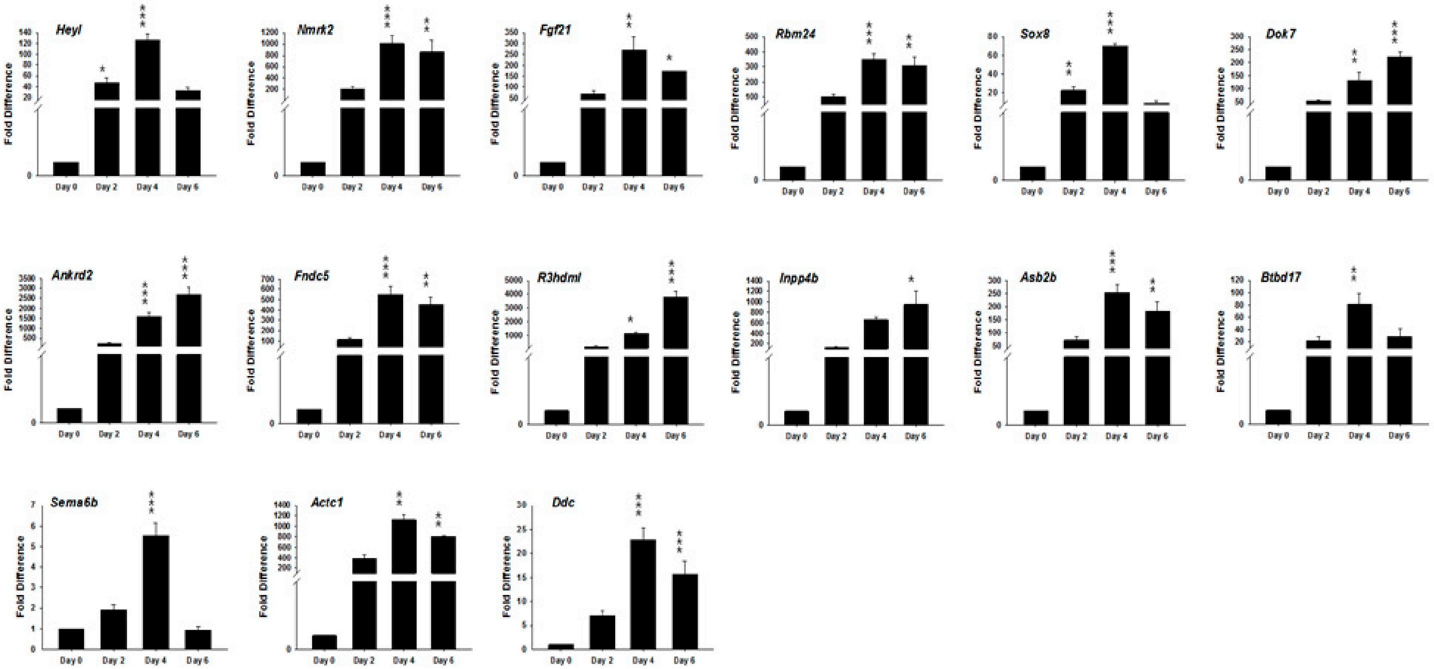

Supplementary Fig. 2. Time-course study of down-regulated genes during myoblast differentiation. Expression of down-regulated genes at each time from the start of differentiation (Day 0) to terminal differentiation (Day 6). Within the same interval, mRNA level at four different times were analyzed by real-time RT-PCR. Day 0 was used as the basement expression level in order to compare the various times. Means±SEM. *p≤0.05, **p≤0.001, ***p≤0.001.

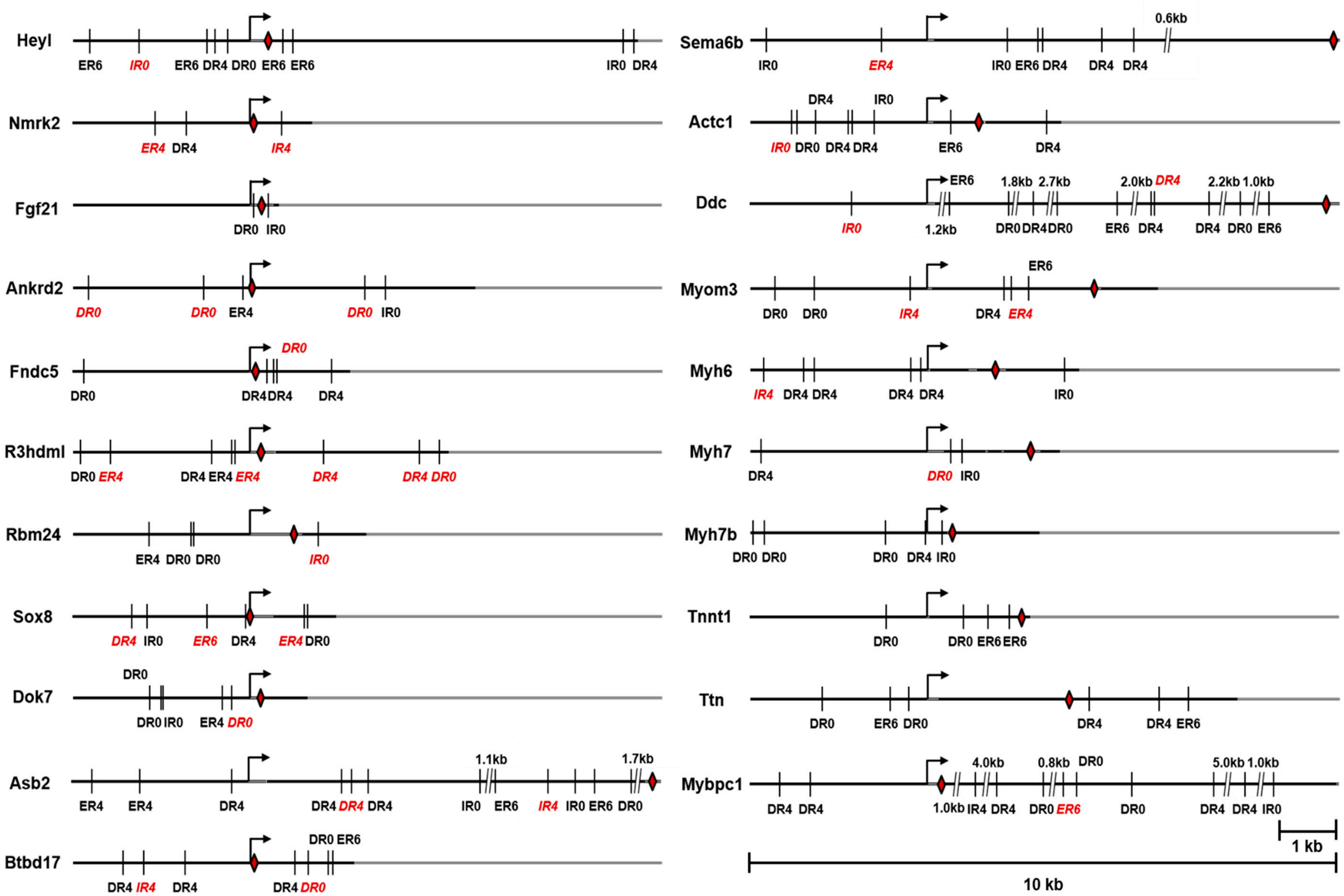
Heyl
ER6 IR0 ER6 DR4 DR0 ER6 ER6 IR0 DR4
Nmrk2
ER4 DR4 IR4
Fgf21
DR0 IR0
Ankrd2
DR0 DR0 ER4 DR0 IR0
Fndc5
DR0
DR0 DR4 DR4 DR4
R3hdml
DR0 ER4 DR4 ER4 ER4 DR4 DR4 DR0
Rbm24
ER4 DR0 DR0 IR0
Sox8
DR4 IR0 ER6 DR4 ER4 DR0
Dok7
DR0
DR0 IR0 ER4 DR0
Asb2
1.1kb 1.7kb
ER4 ER4 DR4 DR4 DR4 DR4 IR0 ER6 IR4 IR0 ER6 DR0
Btbd17
DR0 ER6
DR4 IR4 DR4 DR4 DR0
Sema6b
0.6kb
IR0 ER4 IR0 ER6 DR4 DR4 DR4
Actc1
DR4 IR0
IR0 DR0 DR4 DR4 ER6 DR4
Ddc
ER6 1.8kb 2.7kb 2.0kb DR4 2.2kb 1.0kb
IR0 1.2kb DR0 DR4 DR0 ER6 DR4 DR4 DR0 ER6
Myom3
ER6
DR0 DR0 IR4 DR4 ER4
Myh6
IR4 DR4 DR4 DR4 DR4 IR0
Myh7
DR4 DR0 IR0
Myh7b
DR0 DR0 DR0 DR4 IR0
Tnnt1
DR0 DR0 ER6 ER6
Ttn
DR0 ER6 DR0 DR4 DR4 ER6
Mybpc1
4.0kb 0.8kb DR0 5.0kb 1.0kb
DR4 DR4 1.0kb IR4 DR4 DR0 ER6 DR0 DR4 DR4 IR0
1 kb
10 kb

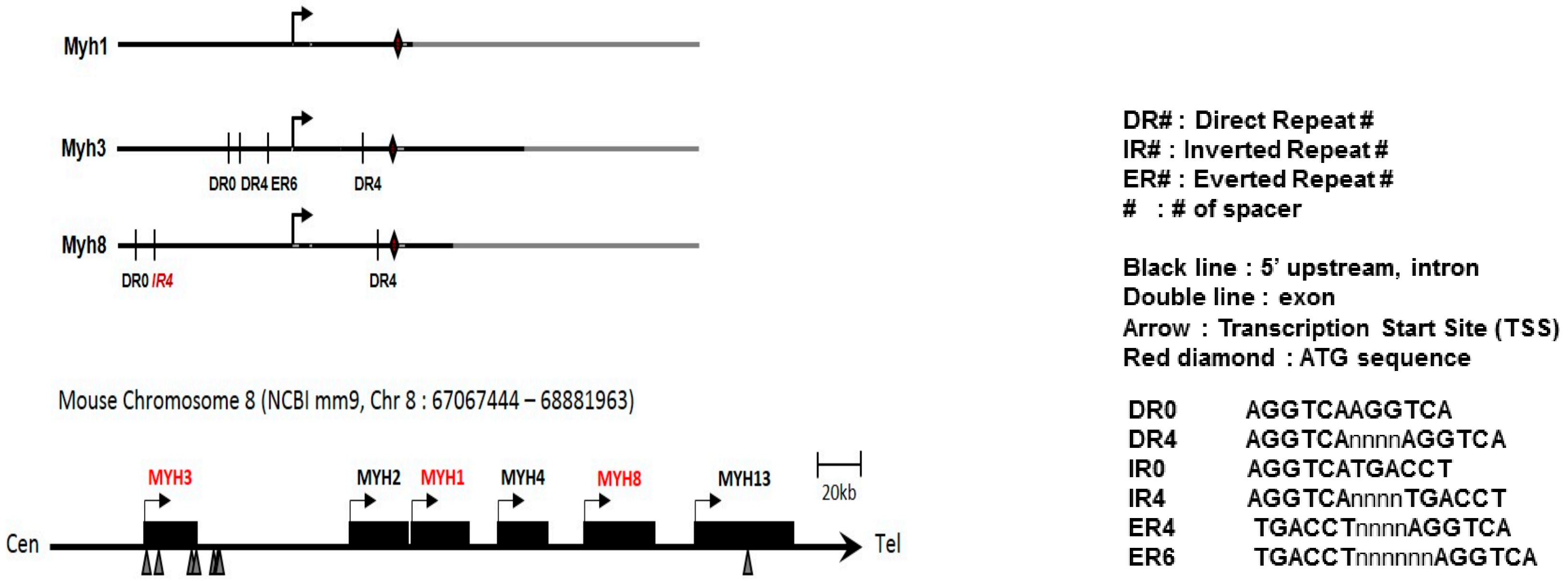

Supplementary Fig. 3. Promoter of down-regulated genes was analyzed to predict TRE binding site. Putative TRE site of down-regulated genes (Black line: 5' upstream, intron, double line: exon, arrow: transcription start site, red diamond: ATG sequence).

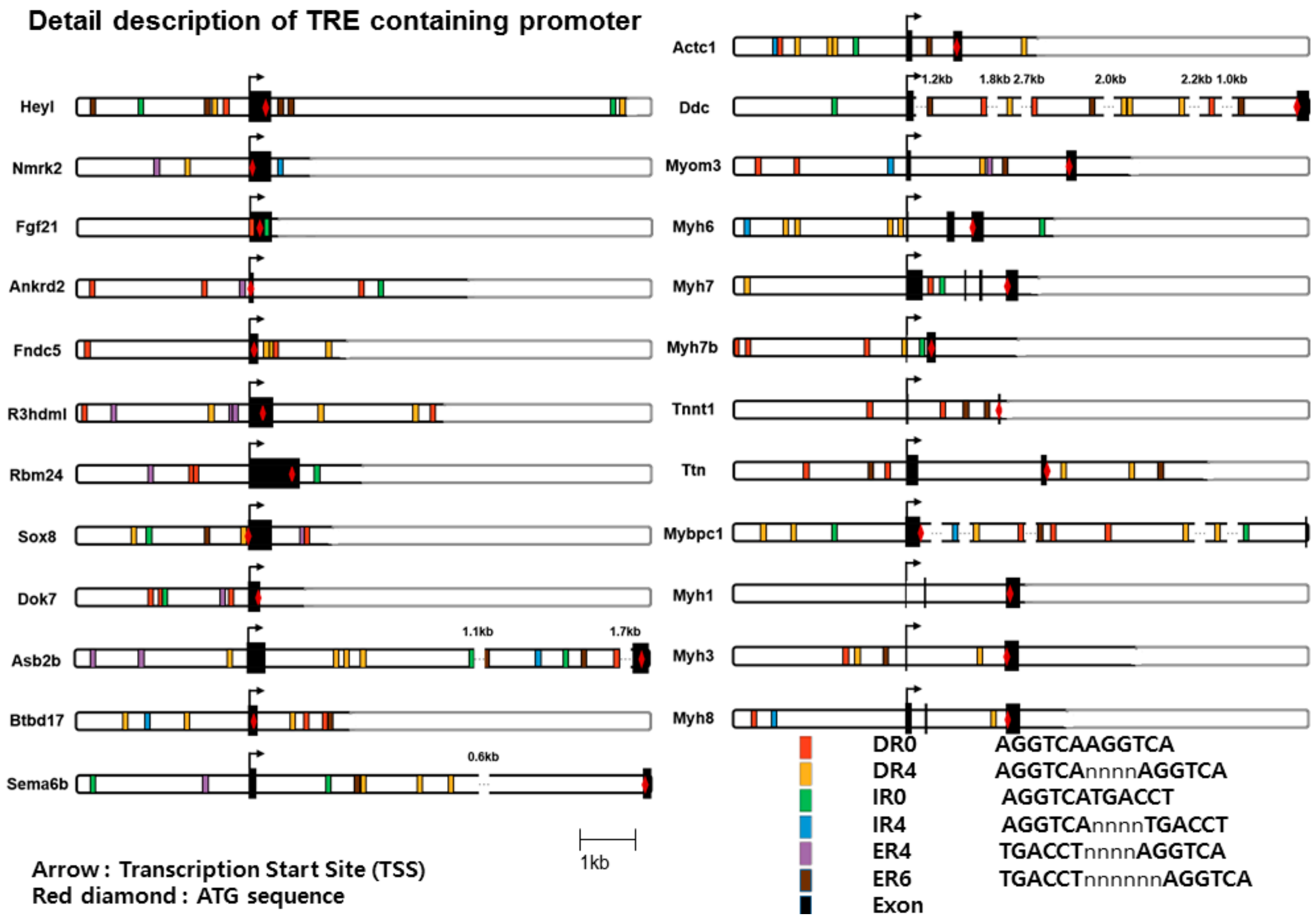

Supplementary Fig. 4. Promoter of down-regulated genes was analyzed to predict TRE binding site. Putative TRE site of down-regulated genes (Arrow: transcription start site, red diamond: ATG sequence).
